# Russian Doll-like 3d–4f Cluster Wheels with Slow Relaxation of Magnetization †

**DOI:** 10.3390/molecules28155906

**Published:** 2023-08-06

**Authors:** Lan Liu, Panpan Yang, Zhihui Qiu, Kai Wang, Dongcheng Liu, Yuning Liang, Huancheng Hu, Huahong Zou, Fupei Liang, Zilu Chen

**Affiliations:** 1 School of Chemistry and Pharmaceutical Sciences, Guangxi Normal University, Guilin 541004, China; 2 Guangxi Key Laboratory of Electrochemical and Magnetochemical Functional Materials, College of Chemistry and Bioengineering, Guilin University of Technology, Guilin 541004, China

**Keywords:** 3d-4f cluster, wheel, slow magnetic relaxation, Russian doll

## Abstract

The solvothermal reactions of LnCl_3_·6H_2_O and MCl_2_·6H_2_O (M = Co, Ni) with 2,2′-diphenol (H_2_L^1^) and 5,7-dichloro-8-hydroxyquinoline (HL^2^) gave three 3d–4f heterometallic wheel-like nano-clusters [Ln_7_M_6_(L^1^)_6_(L^2^)_6_(*µ*_3_-OH)_6_(OCH_3_)_6_Cl(CH_3_CN)_6_]Cl_2_·*x*H_2_O (Ln = Dy, M = Co, *x* = 3 for **1**; Ln = Dy, M = Ni, *x* = 0 for **2**; Ln = Tb, M = Ni, *x* = 0 for **3**) with similar cluster structure. The innermost Ln(III) ion is encapsulated in a planar Ln_6_ ring which is further embedded in a chair-conformation M_6_ ring, constructing a Russian doll-like 3d–4f cluster wheel Ln(III)⸦Ln_6_⸦M_6_. **2** and **3** show obvious slow magnetic relaxation behavior with negligible opening of the magnetic hysteresis loop. Such a Russian doll-like 3d–4f cluster wheel with the lanthanide disc isolated by transition metallo-ring is rarely reported.

## 1. Introduction

Research on 3d–4f single-molecule magnets (SMMs) has mushroomed since the first report of 3d–4f SMM by Matsumoto’s group in 2004 [1,2,3,4,5]. The fundamental interest in SMMs is the potential applications in molecular spintronics and nanoscale magnetic devices [6,7]. As we all know, lanthanide ions such as Dy(III) and Tb(III) have a high-spin ground state and large uniaxial magnetic anisotropy [6,8,9,10,11,12]. In 2021, two chiral Dy(III) macrocyclic complexes with a record anisotropy barrier exceeding 1800 K and a relaxation time approaching 2500 s at 2.0 K for all known air-stable SMMs were obtained. The nearly perfect axiality of the ground Kramers doublet (KD) enables the open hysteresis loops up to 20 K in the magnetically diluted sample [13]. Different from 4f ions, the intrinsic crystal field effects of transition metal ions are strong but with much weaker spin–orbit coupling, which limits the effective orbital contribution of a single transition metal ion and leads to weak magnetic anisotropy [14,15,16,17,18]. Therefore, the magnetic anisotropies of 3d–4f SMMs are largely contributed by 4f ions. However, the paramagnetic 3d metal ions such as Co^2+^ and Mn^3+^ can help to effectively regulate magnetic exchange in 3d–4f SMMs [19,20,21,22]. Thus, the combination of 3d–4f metal ions can not only achieve high ground state spin and strong magnetic anisotropy, but also enhance the magnetic exchange between metal ions, which is facile for obtaining SMMs with superior performance.

In the past decade, a lot of efforts have been made in the development of 3d–4f SMMs, achieving rich and colorful structures with the aim to improve SMM performances. In 2007, a four-shell, nesting doll-like 3d–4f cluster containing 108 metal ions was reported. The metal ions are arranged into an unprecedented four-shell nesting doll-like structure of Ni(II)_6_Gd_2_(III)⸦Gd_20_(III)⸦Gd_32_(III)⸦Ni(II)_48_ to generate a giant cluster [23]. In 2020, the group of Jun-Liang Liu reported one Dy-NC-Fe-CN-Dy SMM and one Dy-NC-Co-CN-Dy SMM with a record barrier of 659 and 975 K among d–f SMMs [24]. However, the performances of these reported SMMs are still far from expected. Furthermore, there are a lot of other issues to be solved, such as the effective regulation and enhancement of magnetic coupling between 3d and 4f metal ions and the explanation of the relaxation mechanism [25,26,27,28]. Therefore, it is still of great significance to design and synthesize more 3d–4f SMMs with novel structures to improve SMM performances and to understand the magneto–structural correlation.

As well known, the performances of these 3d–4f SMMs are closely correlated with their structures including the coordination geometry of single lanthanide ion which tunes the anisotropy of single lanthanide ion, as well as the topological arrangement of metal ions in the cluster which adjusts the synergistic effect of metal ions and intermetallic interactions in the cluster. Ligands play an important role in obtaining expected aesthetic structures [29,30,31,32,33,34], which requires the appropriate selection of bridging groups for tuning intermetallic interactions, spacers between the bridging groups for further adjusting the intermetallic interactions through changing the intermetallic distances, and non-coordinating groups for controlling the growth of targeted clusters in dimensionalities.

**Scheme 1:**
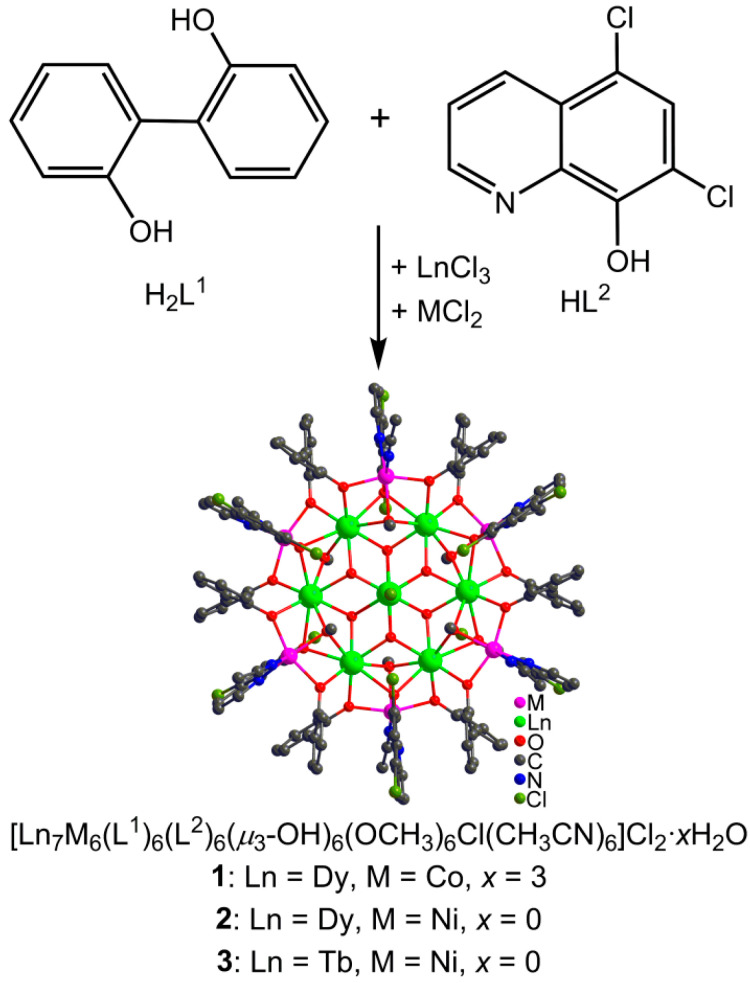
Preparation of **1**–**3**.

With an overall consideration of all the above-mentioned information, we selected two ligands of 2,2′-diphenol (H_2_L^1^) and 5,7-dichloro-8-hydroxyquinoline (HL^2^) to synergistically construct 3d-4f clusters as depicted in Scheme 1. We succeeded in obtaining three 3d–4f heterometallic wheel-like nano-clusters [Ln_7_M_6_(L^1^)_6_(L^2^)_6_(*µ*_3_-OH)_6_(OCH_3_)_6_Cl-(CH_3_CN)_6_]Cl_2_·*x*H_2_O (Ln = Dy, M = Co, *x* = 3 for **1**; Ln = Dy, M = Ni, *x* = 0 for **2**; Ln = Tb, M = Ni, *x* = 0 for **3**), which show similar Russian doll-like 3d-4f wheel skeleton of Ln(III)⸦Ln_6_⸦M_6_. Their structures and magnetic properties were investigated in detail.

## 2. Results and Discussion

**Figure 1:**
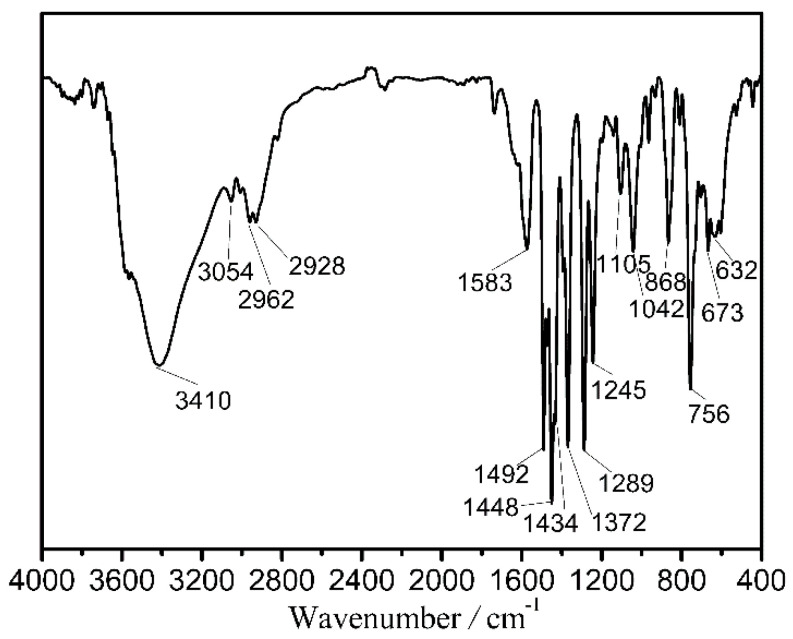
Infrared spectrum of **1**.

**Figure 2:**
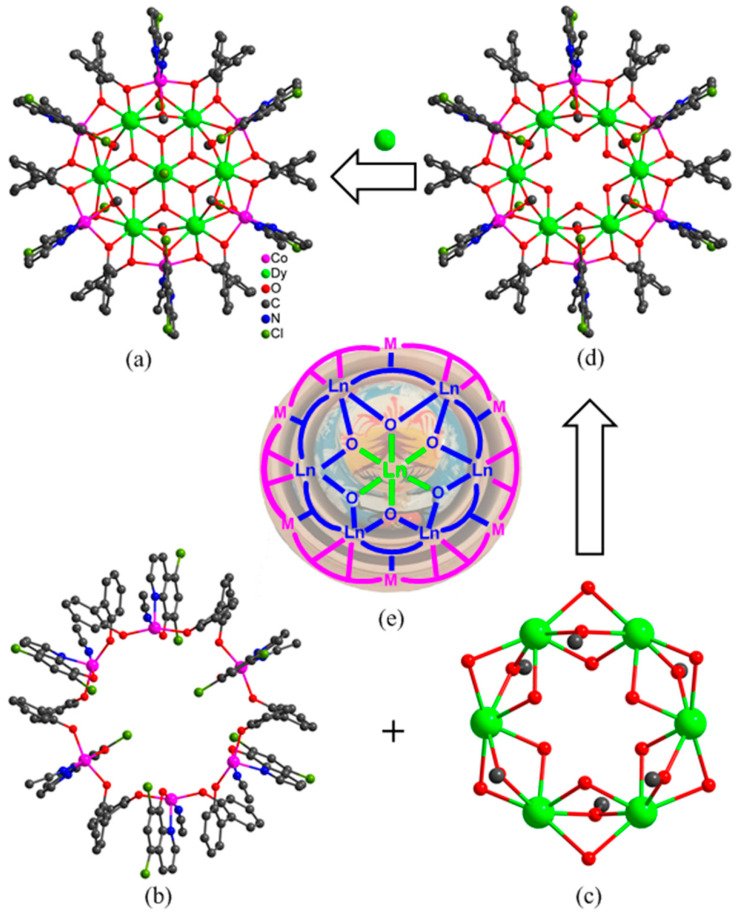
Molecular structure (**a**), peripheral Co_6_ ring (**b**), inner Dy_6_ ring (**c**), Co_6_Dy_6_ metallacrown ether (**d**) and a show of the Russian doll-like skeleton of “Dy(III)⸦Dy_6_⸦Co_6_” (**e**) in **1**.

The sample purities for the titled clusters were confirmed by their experimental powder X-ray diffraction (PXRD) curves which fit well with their simulated ones from their structures as shown in Figure S1. The infrared spectra of **1**–**3** were recorded, which display similar features. Thus, only the infrared spectrum of **1** is presented in Figure 1 and discussed here in detail with the infrared spectra of **2** and **3** shown in Figure S2. It shows bands at 1583, 1492, 1434, 1372, 1289, and 1245 cm^−1^ for C=C and C–C stretching vibrations of the quinoline and diphenyl skeletons, 1448 cm^−1^ for C=N stretching vibration, 1105 cm^−1^ for C–O stretching vibration, and 1042 and 868 cm^−1^ for C–Cl stretching vibration [35]. This information supports the composition of **1**. The structures of titled clusters were determined from single crystal X-ray diffraction analysis with related data shown in Figure 2, Figures S3 and S4 and Tables S1–S4.

### 2.1. Crystal Structures

The structural analysis results revealed a space group of *P*a-3 (cubic system) for **1**, and *R*-3 (trigonal system) for **2** and **3**. But they present similar composition and structure with the formula of [Ln_7_M_6_(L^1^)_6_(L^2^)_6_(*µ*_3_-OH)_6_(OCH_3_)_6_Cl(CH_3_CN)_6_]Cl_2_·*x*H_2_O (Ln = Dy, M = Co, *x* = 3 for **1**; Ln = Dy, M = Ni, *x* = 0 for **2**; Ln = Tb, M = Ni, *x* = 0 for **3**), in which seven Ln(III) ions and six octahedral M(II) ions are linked by six (L^1^)^−^, six (L^2^)^2−^, six OH^−^, and six CH_3_O^−^ ligands. Thus, only the structure of **1** was discussed here in detail as an example for further understanding the structural features of the titled 3d–4f heterometallic wheel-like nano-clusters as shown in Figure 2a. Dy1 in **1** is eight-coordinated in a square antiprism geometry as revealed by the SHAPE calculation with the eight O atoms provided by two OH^−^, two CH_3_O^−^, one two (L^1^)^2−^, and two (L^2^)^−^ ligands. The central Dy2 ion in **1** is disordered over two symmetry-related positions in an occupancy ratio of 0.5:0.5. The Co(II) ions in **1** show severely distorted octahedral geometries with the two N and four O atoms from one CH_3_CN, one CH_3_O^−^, one (L^2^)^−^ and two (L^1^)^2−^ ligands. Each (L^1^)^2−^ ligand bridges one Dy(III) and two Co(II) ions with each of its two phenolic O atoms linking one Dy(III) and one Co(II) ion, presenting a bridging mode of *μ*_3_-η^1^:η^1^:η^2^. Each (L^2^)^−^ ligand chelates one Co(II) ion and uses its phenolic O atom to link this Co(II) ion and two Dy(III) ions, showing a bridging mode of *μ*_3_−η^1^:η^1^:η^2^.

The coordination unit of [Dy_7_Co_6_(L^1^)_6_(L^2^)_6_(*µ*_3_-OH)_6_(OCH_3_)_6_Cl(CH_3_CN)_6_]^2+^ can be divided into three shells. The first shell is the peripheral Co_6_ ring (Figure 2b) which is formed from six Co(II) ions linked by six (L^1^)^2−^ ligands with each Co(II) further chelated by one (L^2^)^2−^. The second shell is the Dy_6_ ring (Figure 2c), in which each neighboring two Dy(III) ions are triply bridged by three O atoms from one OH^−^, one CH_3_O^−,^ and one (L^2^)^−^ ligand. The Co_6_ and Dy_6_ rings are consolidated through six (L^1^)^2−^ and six (L^2^)^2−^ ligands, leading to the construction of a Russian doll-like Co_6_Dy_6_ metallacrown ether (Figure 2d) with the Dy_6_ ring embedded in the Co_6_ ring. Interestingly, the Dy_6_ ring presents a planar conformation; however, the Co_6_ ring shows a chair conformation as depicted in Figure 2e. The third shell is the innermost Dy(III) ion which is disordered over two positions and is situated on the axial position of the *S*_6_ axis in the molecule. It means that the innermost Dy(III) ion is encapsulated by a Dy_6_ metallacrown ether which is further encapsulated by a Co_6_ ring, forming a Russian doll-like 3d–4f Cluster wheel symbolized as “Dy(III)⸦Dy_6_⸦Co_6_” with the inner “Dy(III)⸦Dy_6_” disc protected by a Co_6_ ring. It represents the first kind of Russian doll-like cluster wheel with a lanthanide disc encapsulated in a transition metal ring, although a lot of 3d–4f clusters have already been reported with diverse skeletons such as icosahedron, icosidodecahedron, and ring [36,37,38,39,40].

### 2.2. Magnetic Properties

**Figure 3:**
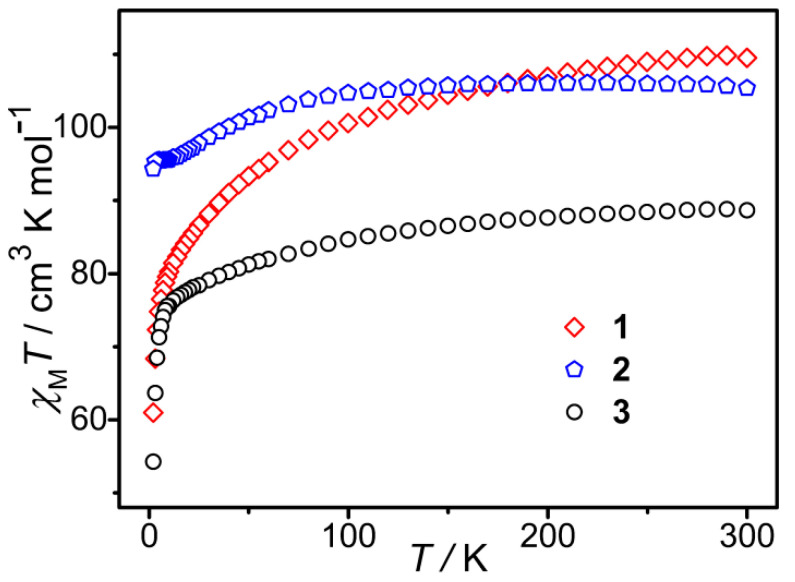
*χ*_m_*T*−*T* curves for **1**–**3**.

Under a direct current (DC) magnetic field of 1000 Oe, the change of molar magnetic susceptibilities of **1**–**3** (Figure 3) with temperature (2–300 K) was measured. The *χ*_m_*T* values of **1**–**3** at 300 K are 109.52, 105.40, and 88.69 cm^3^ K mol^−1^, respectively, which approach the theoretically calculated values of 110.44, 105.19, and 88.74 cm^3^ K mol^−1^ for the corresponding free six transition metal ions (with *g* = 2) and seven lanthanide ions. The value for compound **1** is higher than that expected due to the orbital contribution of the Co(II) ions. With the temperature decreasing, the *χ*_m_*T* values of **1**–**3** decrease slowly in the high-temperature range and then quickly in the low-temperature range, with the lowest values of 61.00, 94.31, and 54.29 cm^3^ K mol^−1^ at 2 K for **1** and **3**, respectively. This kind of behavior revealed in their *χ*_m_*T* versus *T* curves is largely due to thermal depopulation of the Mj sublevels of the lanthanide ions arising from the splitting of the spin–orbit coupling ground term by the ligand crystal field and dipole–dipole interactions between molecules [41].

The isothermal magnetizations of **1**–**3** were measured as a function of field at different temperatures of 2, 3, and 5 K, which were plotted as M versus H and M versus H/T curves (Figures S5–S7). Their magnetizations increase fast at the beginning and then slowly with the increasing field as revealed in both isothermal M versus H and M versus H/T curves. Furthermore, their M versus H/T curves recorded at different temperatures do not overlap in the high H/T range. These features help us to conclude the possible existence of magnetic anisotropy, as well as possible low-lying excited states of their metal ions. The further hysteresis measurements at 2 K for **1**–**3** (Figure S8) revealed just imperceptible hysteresis loops [42,43].

**Figure 4:**
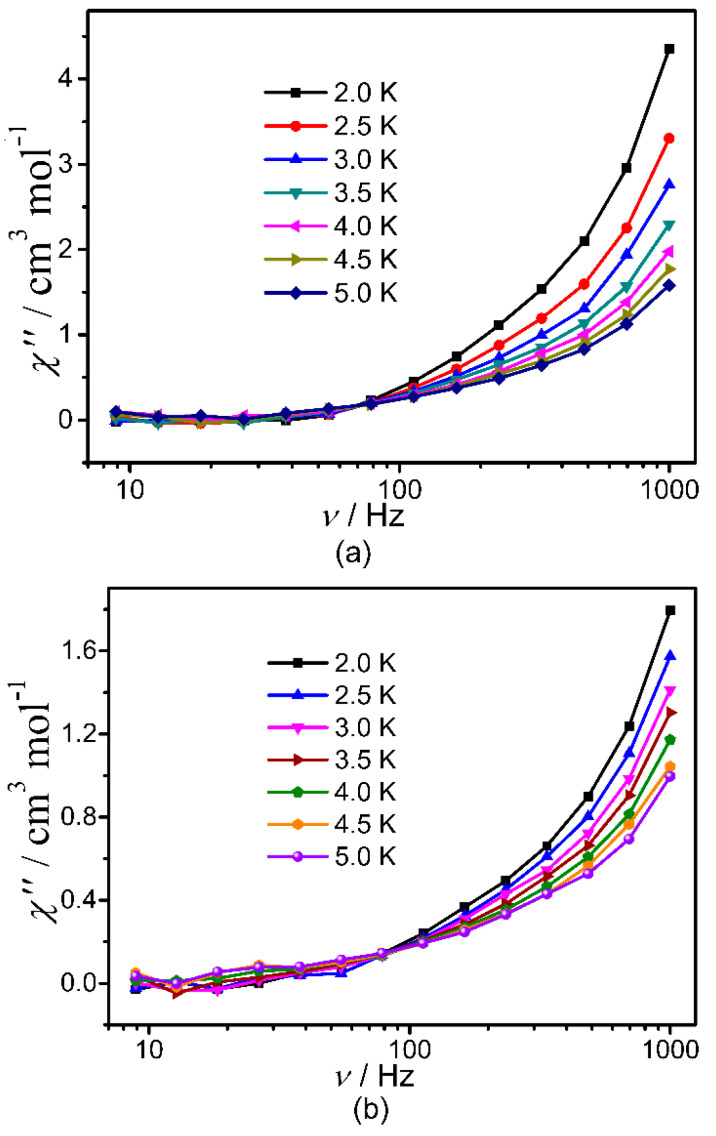
*χ*″−*v* curves for **2** (**a**) and **3** (**b**) under zero-field.

Subsequently, the dynamic magnetic properties of **1**–**3** were studied in detail by measuring their temperature- and frequency-dependent in-phase (*χ*′) and out-of-phase (*χ*″) alternating current (ac) magnetic susceptibilities. It revealed in Figure S9 nearly zero *χ*″ value and obvious temperature dependence for the *χ*′ value with indistinguishable frequency dependency of *χ*′ value for **1** under zero dc field. However, non-zero *χ*″ value and obvious temperature dependence *χ*′ value were observed for **2** and **3** (Figure 4, Figures S10 and S11) under zero dc field together with obvious frequency dependency of *χ*′ value. But no peaks were found for the *χ*″-*v* curves of all title clusters probably due to the quantum tunneling effect (QTM). No appropriate external dc fields were found for the suppression of QTM. Nevertheless, all of these can confirm the presence of slow magnetic relaxation in **2** and **3**. Considering the fact of absence of maximums in their *χ*″-*v* curves, the barriers of **2** and **3** were estimated by fitting their ln(*χ*″/*χ*′) versus 1/*T* curves (Figure S12) based on the equation of ln(*χ*″/*χ*′) = ln(2π*vτ*_0_) + *U*_eff_/*k*_B_*T* [44,45,46], giving *U*_eff_/*k*_B_ ≈ 0.53(8) K and *τ*_0_ ≈ 7.6(2) × 10^−6^ for **2**, and *U*_eff_/*k*_B_ ≈ 0.38(8) K and *τ*_0_ ≈ 8.0(4) × 10^−6^ for **3**.

The magnetic interactions between Ln(III) ions in **2** and **3**, as well as between Ln(III) ion and Ni(II) ion, are presumably weak because the 4f orbitals of Ln(III) ions are deeply buried and shielded by 5p and 6s orbitals and, thus, cannot effectively overlap with the valence orbitals of bridging atoms [47]. Furthermore, the Ni(II) ions in **2** and **3** are well separated by the (L^1^)^2−^ ligand with a separation of 6.0119(1) and 6.0342(1) Å, which does not favor effective magnetic interaction between Ni(II) ions. The weak intermetallic magnetic interaction, together with the coordination symmetries of single metal ions (especially Dy(III) and Tb(III) ion) in **2** and **3**, cannot induce enough coercive fields for suppressing QTM, thus leading to poor magnetic performance of **2** and **3**, such as the negligible opening of magnetic hysteresis loop and low barrier [48,49,50].

## 3. Materials and Methods

### 3.1. General Materials and Methods

All chemical reagents were commercially obtained and used directly. The Fourier transform infrared (FT-IR) data of these complexes were collected on PerkinElmer Spectrum One FT-IR spectrometer using the corresponding KBr Pellets in the wavenumber range of 4000–400 cm*^−^*^1^. The powder X-ray diffraction (PXRD) measurements were carried out on a Rigaku D/max 2500 v/pc diffractometer equipped with Cu-Kα radiation (λ = 1.5418 Å) at 40 kV and 40 mA, with a step size of 0.02° in 2θ and a scan speed of 5 °min*^−^*^1^. Elemental analyses for C, H, and N for the four complexes were performed on an Elementar Micro cube C, H, N elemental analyzer. 

### 3.2. Synthesis

#### 3.2.1. Synthesis of [Dy_7_Co_6_(L^1^)_6_(L^2^)_6_(µ_3_-OH)_6_(OCH_3_)_6_Cl(CH_3_CN)_6_]Cl_2_·3H_2_O (**1**)

A methanol solution (1 mL) of DyCl_3_·6H_2_O (0.05 mmol, 0.0185 g), CoCl_2_·6H_2_O (0.05 mmol, 0.0119 g), H_2_L^1^ (0.1 mmol, 0.0186 g), and an acetonitrile solution (1 mL) of HL^2^ (0.05 mmol, 0.0107 g) were mixed and subsequently added into an 18 cm-long Pyrex tube with one end closed. After the following addition of trimethylamine (0.25 mmol, 35 μL), the Pyrex tube was sealed under vacuum and put in an oven at 90 °C. After a reaction duration of 1 day, it was slowly cooled to room temperature, giving red crystals with a yield of 31% (calculated based on Dy). Elemental analysis (%) (C_144_H_120_N_12_O_33_Cl_15_Co_6_Dy_7_), calculated: C, 37.85, H, 2.65, and N, 3.68. Found: C, 37.53, H, 2.76, and N, 3.85. IR (KBr pellet, cm^−1^, Figure 1): 3410 s, 3054 w, 2926 m, 2928 m, 1583 w, 1492 s, 1448 s, 1434 s, 1372 s, 1289 s, 1245 m, 1105 w, 1042 m, 868 m, 756 s, 673 m, and 632 m.

#### 3.2.2. Synthesis of [Dy_7_Ni_6_(L^1^)_6_(L^2^)_6_(µ_3_-OH)_6_(OCH_3_)_6_Cl(CH_3_CN)_6_]Cl_2_ (**2**)

The synthesis of **2** is similar to that for **1** using NiCl_2_·6H_2_O instead of CoCl_2_·6H_2_O. The yield for **2** is about 22% (calculated on the amount of Dy). Elemental analysis (%) (C_144_H_114_N_12_O_30_Cl_15_Ni_6_Dy_7_), calculated: C, 38.32, H, 2.55, and N, 3.72. Found: C, 37.98, H, 2.85, and N, 3.52. IR (KBr pellet, cm^−1^, Figure S2): 3417 s, 3056 w, 3009 w, 2973 w, 2937 w, 1582 w, 1492 s, 1462 s, 1433 s, 1364 s, 1289 s, 1251 m, 1109 w, 1041 m, 868 m, 748 s, 665 m, and 607 m.

#### 3.2.3. Synthesis of [Tb_7_Ni_6_(L^1^)_6_(L^2^)_6_(µ_3_-OH)_6_(OCH_3_)_6_Cl(CH_3_CN)_6_]Cl_2_ (**3**)

The synthesis of **3** is similar to that for **2** using TbCl_3_·6H_2_O instead of DyCl_3_·6H_2_O. The yield for **3** is about 25% (calculated on the amount of Dy). Elemental analysis (%) (C_144_H_114_N_12_O_30_Cl_15_Ni_6_Tb_7_), calculated: C, 38.53, H, 2.56, and N, 3.74. Found: C, 38.78, H, 2.50, and N, 3.52. IR (KBr pellet, cm^−1^, Figure S2): 3590 w, 3049 w, 3006 w, 2967 w, 2930 w, 2899 w, 2808 w, 2290 w, 1583 w, 1492 s, 1457 s, 1432 s, 1364 s, 1282 s, 1245 m, 1109 w, 1034 m, 868 m, 756 s, 665 m, 635 m, and 605 m.

## 4. Conclusions

In summary, we succeeded in obtaining three similar 3d–4f heterometallic wheel-like Ln_7_M_6_ nano-clusters, in which seven square antiprismatic Dy(III) ions and six octahedral Co(II) ions are linked by six (L^1^)^−^, six (L^2^)^2−^, six OH^−^, and six CH_3_O^−^ ligands. The (L^1^)^2−^ ligand bridges one Dy(III) and two Co(II) ions with each of its two phenolic O atoms linking one Dy(III) and one Co(II) ion. Each (L^2^)^−^ ligand chelates one Co(II) ion and uses its phenolic O atom to link this Co(II) ion and two Dy(III) ions. The most interesting is that the innermost Ln (III) ion is encapsulated in a planar Ln_6_ ring which is further embedded in a chair-conformation M_6_ ring, constructing a Russian doll-like 3d-4f cluster wheel Ln(III)⸦Ln_6_⸦M_6_. This kind of Russian doll-like cluster wheel with a lanthanide disc encapsulated in a transition metal ring is rarely reported. Magnetic tests showed that both **2** and **3** show obvious slow magnetic relaxation behavior with negligible opening of the magnetic hysteresis loop.

## Data Availability

The data presented in this study are available in the article and Supplementary Material. CCDC 2252491, 2252492 and 2252493 for **1**–**3**, respectively, contain the supplementary crystallographic data for this paper, which can be freely accessed via www.ccdc.cam.ac.uk/data_request/cif, or by emailing data_request@ccdc.cam.ac.uk, or by contacting The Cambridge Crystallographic Data Centre, 12 Union Road, Cambridge CB2 1EZ, UK; fax: +44-1223-336033.

## Supplementary Materials

The following supporting information can be downloaded at: https://www.mdpi.com/article/10.3390/molecules28155906/s1. Figure S1. (a), (b), (c) correspond to the powder diffraction patterns of complexes **1**–**3**, respectively. Figure S2. Infrared spectra of complexes **2** (left) and **3** (right). Figure S3. (a) Coordination mode diagram of Dy(III); (b) Coordination mode diagram of Co(II); (c) Ligand (L^1^)^2−^ coordination pattern diagram; (d) Coordination pattern of ligand (L^2^)^−^. Figure S4. Metal skeleton of Co_6_Dy_6_ metallacrown ether in **1**. Figure S5. M-H (a) and M-HT^−1^ (b) curves of **1**. Figure S6. M-H (a) and M-HT^−1^ (b) curves of **2**. Figure S7. M-H (a) and M-HT^−1^ (b) curves of **3**. Figure S8. Hysteresis curves measured at 2 K for **1** (a), **2** (b), **3** (c) with the scan rate of 200 Oe/s. Figure S9. Temperature-dependent (a) and frequency-dependent (b) ac magnetic susceptibilities under zero dc field for **1**. Figure S10. Temperature-dependent (a) and frequency-dependent (b) ac magnetic susceptibilities under zero dc field for **2**. Figure S11. Temperature-dependent (a) and frequency-dependent (b) ac magnetic susceptibilities under zero dc field for **3**. Figure S12. (a) and (b) correspond to ln(*χ*″/*χ*′) versus 1/T plots for complexes **2** and **3**, respectively. Table S1. Crystal data and structural refining parameters for **1**–**3**. Table S2 Selected bond lengths (Å) and angles (º) for complex **1**. Table S3 Selected bond lengths (Å) and angles (º) for complex **2**. Table S4 Selected bond lengths (Å) and angles (º) for complex **3**.
