# Supplementary material for: Russian Doll-like 3d–4f Cluster Wheels with Slow Relaxation of Magnetization
Source: Molecules. 2023 Aug 6;28(15):5906. doi: 10.3390/molecules28155906 (PMC10421525; doi:10.3390/molecules28155906)

checkCIF/PLATON report

Structure factors have been supplied for datablock(s) 106-Pa-3

THIS REPORT IS FOR GUIDANCE ONLY. IF USED AS PART OF A REVIEW PROCEDURE FOR PUBLICATION, IT SHOULD NOT REPLACE THE EXPERTISE OF AN EXPERIENCED CRYSTALLOGRAPHIC REFEREE.

No syntax errors found. CIF dictionary Interpreting this report

Datablock: 106-Pa-3

```
Bond precision:   C-C = 0.0068 A           Wavelength=0.71073

Cell:              a=25.0221(2)      b=25.0221(2)      c=25.0221(2)
                   alpha=90          beta=90           gamma=90
Temperature:       153 K
```

	Calculated	Reported
Volume	15666.5(4)	15666.5(4)
Space group	P a -3	P a -3
Hall group	-P 2ac 2ab	-P 2ac 2ab
Moiety formula	C144 H114 Cl13 Co6 Dy7 N12 O30, 2(Cl), 3(H2 O)	C144 H114 Cl13 Co6 Dy7 N12 O30, 2(Cl), 3(H2 O)
Sum formula	C144 H120 Cl15 Co6 Dy7 N12 O33	C144 H120 Cl15 Co6 Dy7 N12 O33
Mr	4569.42	4569.34
Dx,g cm-3	1.937	1.937
Z	4	4
Mu (mm-1)	4.240	4.240
F000	8844.1	8844.0
F000'	8859.18	
h,k,lmax	29,29,29	29,29,29
Nref	4615	4622
Tmin,Tmax	0.357,0.466	0.365,0.474
Tmin'	0.319	

```
Correction method= # Reported T Limits: Tmin=0.365 Tmax=0.474
AbsCorr = MULTI-SCAN

Data completeness= 1.002            Theta(max)= 25.007
```

R(reflections)= 0.0377(3962) wR2(reflections)= 0.1108(4622)

S = 1.069 Npar= 314

The following ALERTS were generated. Each ALERT has the format
test-name_ALERT_alert-type_alert-level.
Click on the hyperlinks for more details of the test.

Alert level C

```
PLAT220_ALERT_2_C NonSolvent   Resd 1 Cl   Ueq(max)/Ueq(min) Range        4.1 Ratio
PLAT230_ALERT_2_C Hirshfeld Test Diff for    O3       --C13      .        5.7 s.u.
PLAT242_ALERT_2_C Low    'MainMol' Ueq as Compared to Neighbors of        C14 Check
PLAT260_ALERT_2_C Large Average Ueq of Residue Including        O7      0.117 Check
PLAT303_ALERT_2_C Full Occupancy Atom H13B      with # Connections       1.11 Check
PLAT369_ALERT_2_C Long   C(sp2)-C(sp2) Bond  C6       - C7       .       1.53 Ang.
PLAT701_ALERT_1_C Bond    Calc   1.6252(7), Rep    1.626(2), Dev..       1.14 Sigma
              DY2      -DY2             1_555  13_655 ........     #   20 Check
PLAT972_ALERT_2_C Check Calcd Resid. Dens.  0.81Ang From Dy2            -1.70 eA-3
PLAT975_ALERT_2_C Check Calcd Resid. Dens.  1.07Ang From O7      .       1.21 eA-3
```

Alert level G

```
PLAT002_ALERT_2_G Number of Distance or Angle Restraints on AtSite          8 Note
PLAT003_ALERT_2_G Number of Uiso or Uij Restrained non-H Atoms ...          8 Report
PLAT007_ALERT_5_G Number of Unrefined Donor-H Atoms .............           4 Report
PLAT068_ALERT_1_G Reported F000 Differs from Calcd (or Missing)...     Please Check
PLAT083_ALERT_2_G SHELXL Second Parameter in WGHT  Unusually Large      91.85 Why ?
PLAT171_ALERT_4_G The CIF-Embedded .res File Contains EADP Records          1 Report
PLAT172_ALERT_4_G The CIF-Embedded .res File Contains DFIX Records          3 Report
PLAT177_ALERT_4_G The CIF-Embedded .res File Contains DELU Records          2 Report
PLAT178_ALERT_4_G The CIF-Embedded .res File Contains SIMU Records          2 Report
PLAT186_ALERT_4_G The CIF-Embedded .res File Contains ISOR Records          3 Report
PLAT300_ALERT_4_G Atom Site Occupancy of Dy2        Constrained at        0.5 Check
PLAT300_ALERT_4_G Atom Site Occupancy of O5         Constrained at        0.5 Check
PLAT300_ALERT_4_G Atom Site Occupancy of O5'        Constrained at        0.5 Check
PLAT300_ALERT_4_G Atom Site Occupancy of H51        Constrained at        0.5 Check
PLAT300_ALERT_4_G Atom Site Occupancy of H52        Constrained at        0.5 Check
PLAT300_ALERT_4_G Atom Site Occupancy of O7         Constrained at        0.5 Check
PLAT300_ALERT_4_G Atom Site Occupancy of H71        Constrained at        0.5 Check
PLAT300_ALERT_4_G Atom Site Occupancy of H72        Constrained at        0.5 Check
PLAT301_ALERT_3_G Main Residue  Disorder .............(Resd  1  )          5% Note
PLAT302_ALERT_4_G Anion/Solvent/Minor-Residue Disorder (Resd  3  )       100% Note
PLAT304_ALERT_4_G Non-Integer Number of Atoms in ..... (Resd  2  )       0.33 Check
PLAT304_ALERT_4_G Non-Integer Number of Atoms in ..... (Resd  3  )       1.50 Check
PLAT480_ALERT_4_G Long H...A H-Bond Reported H16      ..CL4      .       2.91 Ang.
PLAT764_ALERT_4_G Overcomplete CIF Bond List Detected (Rep/Expd) .       1.15 Ratio
PLAT779_ALERT_4_G Suspect or Irrelevant (Bond) Angle(s) in CIF ...      43.70 Deg.
              DY2  -O5   -DY2     1_555   1_555  13_655 ......     #  236 Check
PLAT794_ALERT_5_G Tentative Bond Valency for Co1       (II)      .       2.11 Info
PLAT860_ALERT_3_G Number of Least-Squares Restraints ............          75 Note
PLAT883_ALERT_1_G No Info/Value for _atom_sites_solution_primary .     Please Do !
PLAT909_ALERT_3_G Percentage of I>2sig(I) Data at Theta(Max) Still         75% Note
PLAT910_ALERT_3_G Missing # of FCF Reflection(s) Below Theta(Min).          2 Note
PLAT913_ALERT_3_G Missing # of Very Strong Reflections in FCF ....          2 Note
```

```
PLAT965_ALERT_2_G The SHELXL WEIGHT Optimisation has not Converged     Please Check
PLAT967_ALERT_5_G Note: Two-Theta Cutoff Value in Embedded .res ..       50.0 Degree
PLAT978_ALERT_2_G Number C-C Bonds with Positive Residual Density.          0 Info
PLAT996_ALERT_1_G Non-Standard SHELXL LIST 4 Style FCF Supplied ..          ! Check
```

```
   0 ALERT level A = Most likely a serious problem - resolve or explain
   0 ALERT level B = A potentially serious problem, consider carefully
   9 ALERT level C = Check. Ensure it is not caused by an omission or oversight
  35 ALERT level G = General information/check it is not something unexpected

   4 ALERT type 1 CIF construction/syntax error, inconsistent or missing data
  13 ALERT type 2 Indicator that the structure model may be wrong or deficient
   5 ALERT type 3 Indicator that the structure quality may be low
  19 ALERT type 4 Improvement, methodology, query or suggestion
   3 ALERT type 5 Informative message, check
```

It is advisable to attempt to resolve as many as possible of the alerts in all categories. Often the minor alerts point to easily fixed oversights, errors and omissions in your CIF or refinement strategy, so attention to these fine details can be worthwhile. In order to resolve some of the more serious problems it may be necessary to carry out additional measurements or structure refinements. However, the purpose of your study may justify the reported deviations and the more serious of these should normally be commented upon in the discussion or experimental section of a paper or in the "special_details" fields of the CIF. checkCIF was carefully designed to identify outliers and unusual parameters, but every test has its limitations and alerts that are not important in a particular case may appear. Conversely, the absence of alerts does not guarantee there are no aspects of the results needing attention. It is up to the individual to critically assess their own results and, if necessary, seek expert advice.

Publication of your CIF in IUCr journals

A basic structural check has been run on your CIF. These basic checks will be run on all CIFs submitted for publication in IUCr journals (*Acta Crystallographica*, *Journal of Applied Crystallography*, *Journal of Synchrotron Radiation*); however, if you intend to submit to *Acta Crystallographica Section C* or *E* or *IUCrData*, you should make sure that full publication checks are run on the final version of your CIF prior to submission.

Publication of your CIF in other journals

Please refer to the *Notes for Authors* of the relevant journal for any special instructions relating to CIF submission.

PLATON version of 06/07/2023; check.def file version of 30/06/2023

Datablock 106-Pa-3 - ellipsoid plot

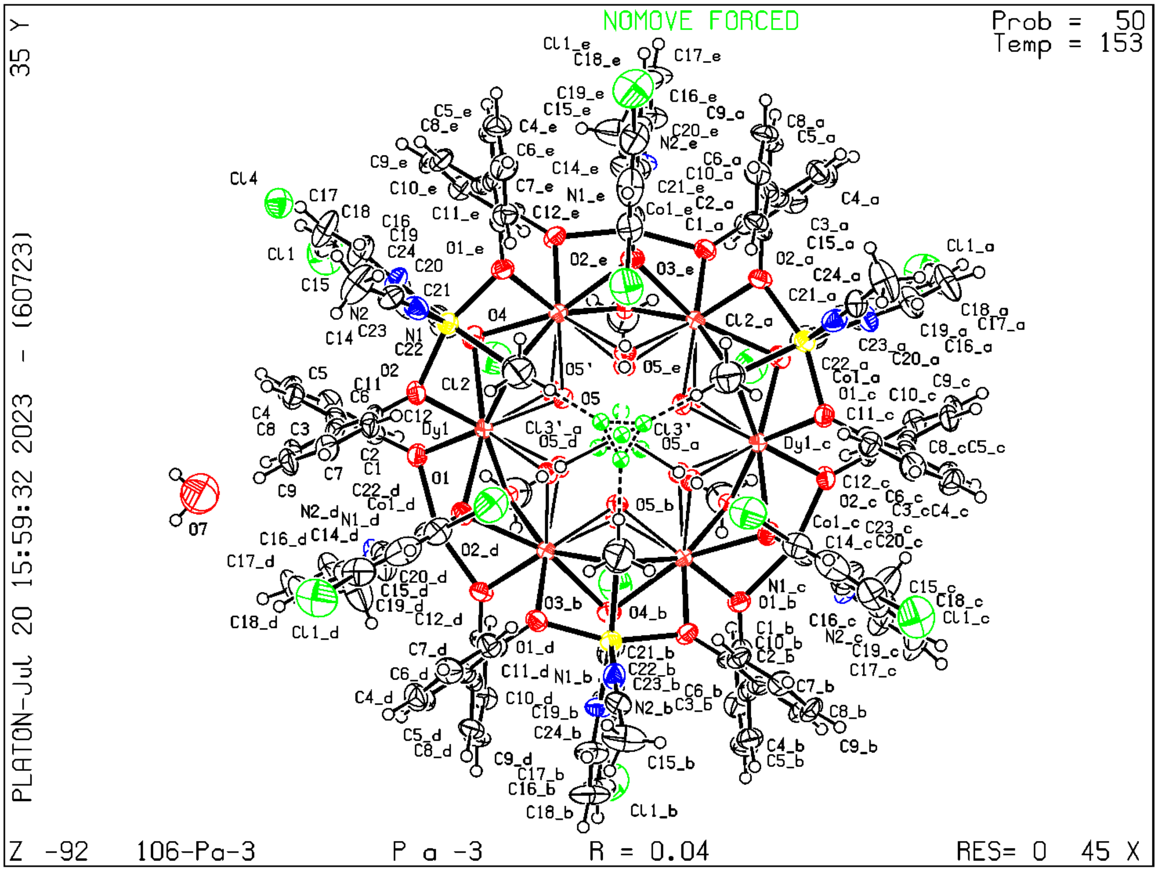

Supplement: Supplementary file 1 [file molecules-28-05906-s001.zip › checkcif of 1.pdf]
