# Supplementary material for: Russian Doll-like 3d–4f Cluster Wheels with Slow Relaxation of Magnetization
Source: Molecules. 2023 Aug 6;28(15):5906. doi: 10.3390/molecules28155906 (PMC10421525; doi:10.3390/molecules28155906)

checkCIF/PLATON report

Structure factors have been supplied for datablock(s) YS-PD-107-100K

THIS REPORT IS FOR GUIDANCE ONLY. IF USED AS PART OF A REVIEW PROCEDURE FOR PUBLICATION, IT SHOULD NOT REPLACE THE EXPERTISE OF AN EXPERIENCED CRYSTALLOGRAPHIC REFEREE.

No syntax errors found. CIF dictionary Interpreting this report

Datablock: YS-PD-107-100K

```
Bond precision:   C-C = 0.0130 A          Wavelength=0.71073

Cell:              a=20.4123(4)      b=20.4123(4)      c=32.2304(8)
                   alpha=90          beta=90           gamma=120
Temperature:       153 K
```

	Calculated	Reported
Volume	11630.0(5)	11630.1(5)
Space group	R -3	R -3 :H
Hall group	-R 3	-R 3
Moiety formula	C144 H114 Cl13 Dy7 N12 Ni6 O30, 2(Cl)	C144 H114 Cl13 Dy7 N12 Ni6 O30, 2(Cl)
Sum formula	C144 H114 Cl15 Dy7 N12 Ni6 O30	C144 H114 Cl15 Dy7 N12 Ni6 O30
Mr	4513.85	4513.98
Dx,g cm-3	1.934	1.934
Z	3	3
Mu (mm-1)	4.368	4.368
F000	6561.0	6561.0
F000’	6572.02	
h,k,lmax	24,24,38	23,24,38
Nref	4568	4561
Tmin,Tmax	0.334,0.476	0.334,0.476
Tmin’	0.309	

```
Correction method= # Reported T Limits: Tmin=0.334 Tmax=0.476
AbsCorr = MULTI-SCAN

Data completeness= 0.998            Theta(max)= 25.011
```

R(reflections)= 0.0489(4074) wR2(reflections)= 0.1383(4561)

S = 1.105 Npar= 395

The following ALERTS were generated. Each ALERT has the format
test-name_ALERT_alert-type_alert-level.
Click on the hyperlinks for more details of the test.

Alert level C

```
PLAT220_ALERT_2_C NonSolvent   Resd 1  C   Ueq(max)/Ueq(min) Range        4.2 Ratio
PLAT220_ALERT_2_C NonSolvent   Resd 1 Cl   Ueq(max)/Ueq(min) Range        4.8 Ratio
PLAT222_ALERT_3_C NonSolvent Resd 1  H   Uiso(max)/Uiso(min) Range        4.9 Ratio
PLAT230_ALERT_2_C Hirshfeld Test Diff for    O3       --C13      .        6.6 s.u.
PLAT232_ALERT_2_C Hirshfeld Test Diff (M-X)  Dy1      --O4_d     .        6.3 s.u.
PLAT234_ALERT_4_C Large Hirshfeld Difference Ni1      --N1       .       0.16 Ang.
PLAT234_ALERT_4_C Large Hirshfeld Difference Cl2      --C22      .       0.16 Ang.
PLAT241_ALERT_2_C High   'MainMol' Ueq as Compared to Neighbors of        C16 Check
PLAT242_ALERT_2_C Low    'MainMol' Ueq as Compared to Neighbors of         O3 Check
PLAT260_ALERT_2_C Large Average Ueq of Residue Including       Cl4      0.200 Check
PLAT260_ALERT_2_C Large Average Ueq of Residue Including      Cl4'      0.199 Check
PLAT303_ALERT_2_C Full Occupancy Atom H13B      with # Connections       1.13 Check
PLAT336_ALERT_2_C Long Bond Distance for ..... C22      -Cl2            1.929 Ang.
PLAT342_ALERT_3_C Low Bond Precision on  C-C Bonds ...............      0.013 Ang.
PLAT420_ALERT_2_C D-H Bond Without Acceptor  Cl3      --H13B     .     Please Check
PLAT911_ALERT_3_C Missing FCF Refl Between Thmin & STh/L=    0.595          4 Report
PLAT934_ALERT_3_C Number of (Iobs-Icalc)/Sigma(W) > 10 Outliers ..          1 Check
PLAT971_ALERT_2_C Check Calcd Resid. Dens.  0.53Ang From Cl2'            1.55 eA-3
PLAT971_ALERT_2_C Check Calcd Resid. Dens.  0.11Ang From Cl3'            1.53 eA-3
```

Alert level G

```
PLAT002_ALERT_2_G Number of Distance or Angle Restraints on AtSite         16 Note
PLAT003_ALERT_2_G Number of Uiso or Uij Restrained non-H Atoms ...         39 Report
PLAT007_ALERT_5_G Number of Unrefined Donor-H Atoms ..............          3 Report
PLAT066_ALERT_1_G Predicted and Reported Tmin&Tmax Range Identical          ? Check
PLAT083_ALERT_2_G SHELXL Second Parameter in WGHT  Unusually Large     281.76 Why ?
PLAT171_ALERT_4_G The CIF-Embedded .res File Contains EADP Records          2 Report
PLAT172_ALERT_4_G The CIF-Embedded .res File Contains DFIX Records          7 Report
PLAT177_ALERT_4_G The CIF-Embedded .res File Contains DELU Records          1 Report
PLAT178_ALERT_4_G The CIF-Embedded .res File Contains SIMU Records          5 Report
PLAT186_ALERT_4_G The CIF-Embedded .res File Contains ISOR Records          2 Report
PLAT188_ALERT_3_G A Non-default SIMU Restraint Value has been used     0.0100 Report
PLAT188_ALERT_3_G A Non-default SIMU Restraint Value has been used     0.0100 Report
PLAT188_ALERT_3_G A Non-default SIMU Restraint Value has been used     0.0100 Report
PLAT192_ALERT_3_G A Non-default DELU Restraint Value for First Par     0.0200 Report
PLAT230_ALERT_2_G Hirshfeld Test Diff for    Cl2'     --C22      .        6.0 s.u.
PLAT300_ALERT_4_G Atom Site Occupancy of Dy2        Constrained at        0.5 Check
PLAT300_ALERT_4_G Atom Site Occupancy of Cl2        Constrained at        0.5 Check
PLAT300_ALERT_4_G Atom Site Occupancy of Cl2'       Constrained at        0.5 Check
PLAT300_ALERT_4_G Atom Site Occupancy of Cl1        Constrained at     0.3333 Check
PLAT300_ALERT_4_G Atom Site Occupancy of Cl1'       Constrained at     0.3333 Check
PLAT300_ALERT_4_G Atom Site Occupancy of Cl3        Constrained at     0.1333 Check
PLAT300_ALERT_4_G Atom Site Occupancy of Cl3'       Constrained at        0.1 Check
PLAT300_ALERT_4_G Atom Site Occupancy of Cl12       Constrained at     0.3333 Check
```

```
PLAT300_ALERT_4_G Atom Site Occupancy of O5         Constrained at        0.5 Check
PLAT300_ALERT_4_G Atom Site Occupancy of O5'        Constrained at        0.5 Check
PLAT300_ALERT_4_G Atom Site Occupancy of H51        Constrained at        0.5 Check
PLAT300_ALERT_4_G Atom Site Occupancy of H52        Constrained at        0.5 Check
PLAT300_ALERT_4_G Atom Site Occupancy of Cl4        Constrained at     0.1667 Check
PLAT300_ALERT_4_G Atom Site Occupancy of Cl4'       Constrained at     0.1667 Check
PLAT301_ALERT_3_G Main Residue  Disorder ..............(Resd  1  )        19% Note
PLAT302_ALERT_4_G Anion/Solvent/Minor-Residue Disorder (Resd  2  )       100% Note
PLAT302_ALERT_4_G Anion/Solvent/Minor-Residue Disorder (Resd  3  )       100% Note
PLAT304_ALERT_4_G Non-Integer Number of Atoms in ..... (Resd  2  )       0.17 Check
PLAT304_ALERT_4_G Non-Integer Number of Atoms in ..... (Resd  3  )       0.17 Check
PLAT413_ALERT_2_G Short Inter XH3 .. XHn     H13A     ..H15A     .       1.98 Ang.
                                          2/3-x,1/3-y,1/3-z  =     13_555 Check
PLAT413_ALERT_2_G Short Inter XH3 .. XHn     H13C     ..H15A     .       2.09 Ang.
                                          2/3-x,1/3-y,1/3-z  =     13_555 Check
PLAT432_ALERT_2_G Short Inter X...Y Contact  C13      ..C15      .       2.96 Ang.
                                          2/3-x,1/3-y,1/3-z  =     13_555 Check
PLAT480_ALERT_4_G Long H...A H-Bond Reported H13C     ..CL2      .       2.90 Ang.
PLAT721_ALERT_1_G Bond    Calc     0.97000, Rep     0.96000 Dev...       0.01 Ang.
              C15      -H15C           1_555   1_555 ........     #   68 Check
PLAT860_ALERT_3_G Number of Least-Squares Restraints ............         322 Note
PLAT883_ALERT_1_G No Info/Value for _atom_sites_solution_primary .     Please Do !
PLAT909_ALERT_3_G Percentage of I>2sig(I) Data at Theta(Max) Still        75% Note
PLAT910_ALERT_3_G Missing # of FCF Reflection(s) Below Theta(Min).          3 Note
PLAT913_ALERT_3_G Missing # of Very Strong Reflections in FCF ....          2 Note
PLAT941_ALERT_3_G Average HKL Measurement Multiplicity ..........         3.3 Low
PLAT965_ALERT_2_G The SHELXL WEIGHT Optimisation has not Converged     Please Check
PLAT967_ALERT_5_G Note: Two-Theta Cutoff Value in Embedded .res ..       50.0 Degree
PLAT978_ALERT_2_G Number C-C Bonds with Positive Residual Density.          2 Info
```

---

0 **ALERT level A** = Most likely a serious problem - resolve or explain
0 **ALERT level B** = A potentially serious problem, consider carefully
19 **ALERT level C** = Check. Ensure it is not caused by an omission or oversight
48 **ALERT level G** = General information/check it is not something unexpected

3 ALERT type 1 CIF construction/syntax error, inconsistent or missing data
22 ALERT type 2 Indicator that the structure model may be wrong or deficient
14 ALERT type 3 Indicator that the structure quality may be low
26 ALERT type 4 Improvement, methodology, query or suggestion
2 ALERT type 5 Informative message, check

It is advisable to attempt to resolve as many as possible of the alerts in all categories. Often the minor alerts point to easily fixed oversights, errors and omissions in your CIF or refinement strategy, so attention to these fine details can be worthwhile. In order to resolve some of the more serious problems it may be necessary to carry out additional measurements or structure refinements. However, the purpose of your study may justify the reported deviations and the more serious of these should normally be commented upon in the discussion or experimental section of a paper or in the "special_details" fields of the CIF. checkCIF was carefully designed to identify outliers and unusual parameters, but every test has its limitations and alerts that are not important in a particular case may appear. Conversely, the absence of alerts does not guarantee there are no aspects of the results needing attention. It is up to the individual to critically assess their own results and, if necessary, seek expert advice.

Publication of your CIF in IUCr journals

A basic structural check has been run on your CIF. These basic checks will be run on all CIFs submitted for publication in IUCr journals (*Acta Crystallographica*, *Journal of Applied Crystallography*, *Journal of Synchrotron Radiation*); however, if you intend to submit to *Acta Crystallographica Section C* or *E* or *IUCrData*, you should make sure that full publication checks are run on the final version of your CIF prior to submission.

Publication of your CIF in other journals

Please refer to the *Notes for Authors* of the relevant journal for any special instructions relating to CIF submission.

PLATON version of 06/07/2023; check.def file version of 30/06/2023

Datablock YS-PD-107-100K - ellipsoid plot

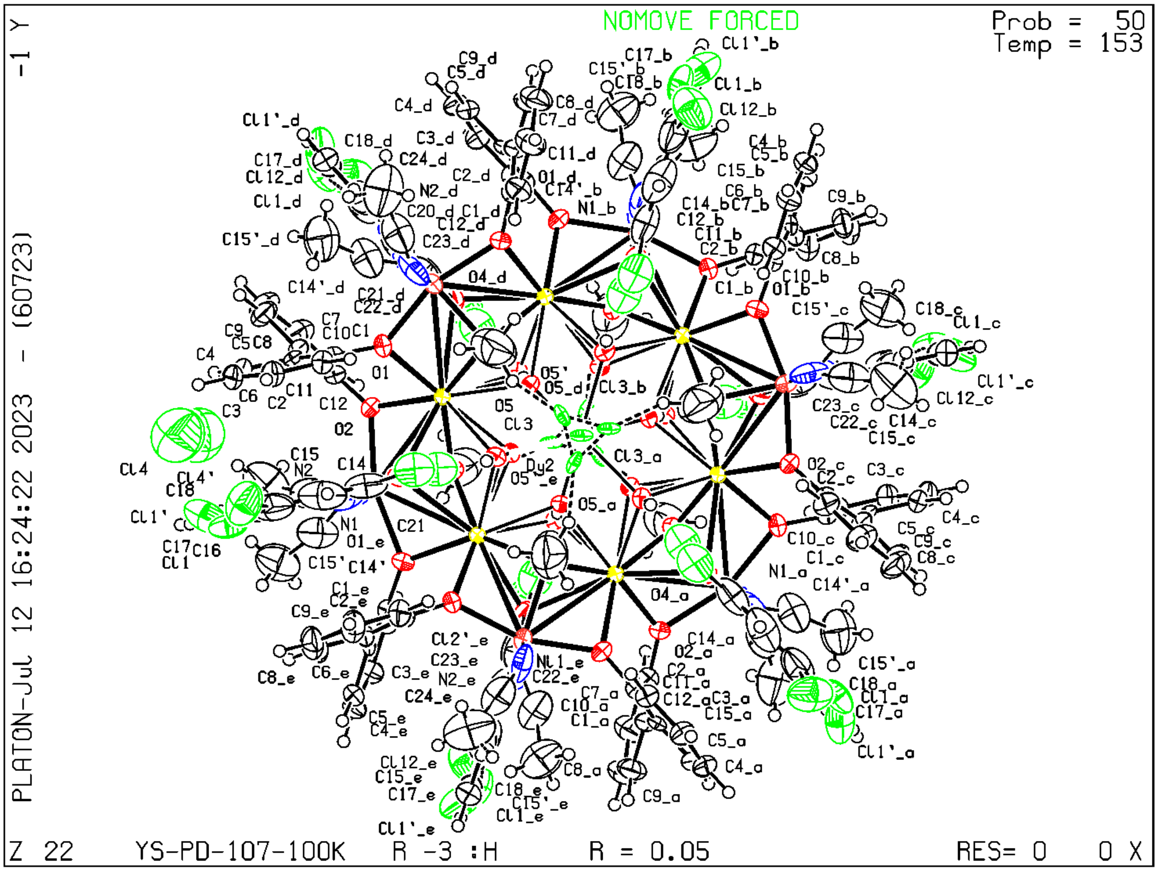

Supplement: Supplementary file 1 [file molecules-28-05906-s001.zip › checkcif of 2.pdf]
