# Supplementary material for: Russian Doll-like 3d–4f Cluster Wheels with Slow Relaxation of Magnetization
Source: Molecules. 2023 Aug 6;28(15):5906. doi: 10.3390/molecules28155906 (PMC10421525; doi:10.3390/molecules28155906)

checkCIF/PLATON report

You have not supplied any structure factors. As a result the full set of tests cannot be run.

THIS REPORT IS FOR GUIDANCE ONLY. IF USED AS PART OF A REVIEW PROCEDURE FOR PUBLICATION, IT SHOULD NOT REPLACE THE EXPERTISE OF AN EXPERIENCED CRYSTALLOGRAPHIC REFEREE.

No syntax errors found. CIF dictionary Interpreting this report

Datablock: 20191011-CZL-PY-2-2

Bond precision:	C-C = 0.0187 A	Wavelength=0.71073	
Cell:	a=20.5601(7)	b=20.5601(7)	c=32.526(3)
	alpha=90	beta=90	gamma=120
Temperature:	153 K		

	Calculated	Reported
Volume	11907.3(14)	11907.2(13)
Space group	R -3	R -3 :H
Hall group	-R 3	-R 3
Moiety formula	C144 H114 Cl13 N12 Ni6 O30 Tb7, 2(Cl)	C144 H114 Cl13 N12 Ni6 O30 Tb7, 2(Cl)
Sum formula	C144 H114 Cl15 N12 Ni6 O30 Tb7	C144 H114 Cl15 N12 Ni6 O30 Tb7
Mr	4488.88	4488.92
Dx,g cm-3	1.878	1.878
Z	3	3
Mu (mm-1)	4.090	4.090
F000	6540.0	6540.0
F000’	6551.35	
h,k,lmax	24,24,38	24,22,38
Nref	4684	4671
Tmin,Tmax	0.374,0.479	0.374,0.479
Tmin’	0.346	

Correction method= # Reported T Limits: Tmin=0.374 Tmax=0.479
AbsCorr = MULTI-SCAN

Data completeness= 0.997 Theta(max)= 25.019

R(reflections)= 0.0595(3601)   wR2(reflections)= 0.1639(4671)

S = 1.038   Npar= 358

The following ALERTS were generated. Each ALERT has the format
test-name_ALERT_alert-type_alert-level.
Click on the hyperlinks for more details of the test.

Alert level C

```
PLAT220_ALERT_2_C NonSolvent   Resd 1  C   Ueq(max)/Ueq(min) Range       5.0 Ratio
PLAT220_ALERT_2_C NonSolvent   Resd 1 Cl   Ueq(max)/Ueq(min) Range       5.4 Ratio
PLAT222_ALERT_3_C NonSolvent Resd 1  H   Uiso(max)/Uiso(min) Range       6.7 Ratio
PLAT230_ALERT_2_C Hirshfeld Test Diff for    O3      --C13      .        5.3 s.u.
PLAT232_ALERT_2_C Hirshfeld Test Diff (M-X)  Ni1     --N1       .        5.7 s.u.
PLAT234_ALERT_4_C Large Hirshfeld Difference N1      --C14      .       0.24 Ang.
PLAT241_ALERT_2_C High   'MainMol' Ueq as Compared to Neighbors of       C14 Check
PLAT242_ALERT_2_C Low    'MainMol' Ueq as Compared to Neighbors of        O3 Check
PLAT242_ALERT_2_C Low    'MainMol' Ueq as Compared to Neighbors of        N1 Check
PLAT242_ALERT_2_C Low    'MainMol' Ueq as Compared to Neighbors of       C22 Check
PLAT260_ALERT_2_C Large Average Ueq of Residue Including      Cl4      0.252 Check
PLAT260_ALERT_2_C Large Average Ueq of Residue Including     Cl4'      0.251 Check
PLAT303_ALERT_2_C Full Occupancy Atom H13C      with # Connections      1.13 Check
PLAT342_ALERT_3_C Low Bond Precision on  C-C Bonds ...............   0.01865 Ang.
PLAT420_ALERT_2_C D-H Bond Without Acceptor  Cl3     --H13C     .     Please Check
```

Alert level G

```
PLAT002_ALERT_2_G Number of Distance or Angle Restraints on AtSite         8 Note
PLAT003_ALERT_2_G Number of Uiso or Uij Restrained non-H Atoms ...        39 Report
PLAT007_ALERT_5_G Number of Unrefined Donor-H Atoms .............          3 Report
PLAT066_ALERT_1_G Predicted and Reported Tmin&Tmax Range Identical         ? Check
PLAT083_ALERT_2_G SHELXL Second Parameter in WGHT  Unusually Large    397.96 Why ?
PLAT172_ALERT_4_G The CIF-Embedded .res File Contains DFIX Records         5 Report
PLAT177_ALERT_4_G The CIF-Embedded .res File Contains DELU Records         3 Report
PLAT178_ALERT_4_G The CIF-Embedded .res File Contains SIMU Records         5 Report
PLAT186_ALERT_4_G The CIF-Embedded .res File Contains ISOR Records         5 Report
PLAT188_ALERT_3_G A Non-default SIMU Restraint Value has been used    0.0100 Report
PLAT188_ALERT_3_G A Non-default SIMU Restraint Value has been used    0.0100 Report
PLAT188_ALERT_3_G A Non-default SIMU Restraint Value has been used    0.0100 Report
PLAT188_ALERT_3_G A Non-default SIMU Restraint Value has been used    0.0100 Report
PLAT192_ALERT_3_G A Non-default DELU Restraint Value for First Par    0.0200 Report
PLAT192_ALERT_3_G A Non-default DELU Restraint Value for First Par    0.0200 Report
PLAT192_ALERT_3_G A Non-default DELU Restraint Value for SecondPar    0.0200 Report
PLAT300_ALERT_4_G Atom Site Occupancy of Tb2        Constrained at       0.5 Check
PLAT300_ALERT_4_G Atom Site Occupancy of Cl3        Constrained at    0.1333 Check
PLAT300_ALERT_4_G Atom Site Occupancy of Cl3'       Constrained at       0.1 Check
PLAT300_ALERT_4_G Atom Site Occupancy of O5         Constrained at       0.5 Check
PLAT300_ALERT_4_G Atom Site Occupancy of O5'        Constrained at       0.5 Check
PLAT300_ALERT_4_G Atom Site Occupancy of H51        Constrained at       0.5 Check
PLAT300_ALERT_4_G Atom Site Occupancy of H52        Constrained at       0.5 Check
PLAT300_ALERT_4_G Atom Site Occupancy of Cl4        Constrained at    0.1667 Check
PLAT300_ALERT_4_G Atom Site Occupancy of Cl4'       Constrained at    0.1667 Check
PLAT301_ALERT_3_G Main Residue  Disorder ..............(Resd  1  )        8% Note
PLAT302_ALERT_4_G Anion/Solvent/Minor-Residue Disorder (Resd  2  )      100% Note
```

```
PLAT302_ALERT_4_G Anion/Solvent/Minor-Residue Disorder (Resd  3  )       100% Note
PLAT304_ALERT_4_G Non-Integer Number of Atoms in ..... (Resd  2  )       0.17 Check
PLAT304_ALERT_4_G Non-Integer Number of Atoms in ..... (Resd  3  )       0.17 Check
PLAT480_ALERT_4_G Long H...A H-Bond Reported H13C     ..O5'      .       2.65 Ang.
PLAT480_ALERT_4_G Long H...A H-Bond Reported H13C     ..CL3      .       2.88 Ang.
PLAT710_ALERT_4_G Delete 1-2-3 or 2-3-4 Linear Torsion Angle ... #         35 Do !
              NI1 -N1  -C14 -C15  -101.00 20.00   1_555   1_555   1_555   1_555
PLAT764_ALERT_4_G Overcomplete CIF Bond List Detected (Rep/Expd) .       1.11 Ratio
PLAT790_ALERT_4_G Centre of Gravity not Within Unit Cell: Resd.  #          3 Note
              Cl
PLAT860_ALERT_3_G Number of Least-Squares Restraints ............         311 Note
PLAT883_ALERT_1_G No Info/Value for _atom_sites_solution_primary .     Please Do !
PLAT941_ALERT_3_G Average HKL Measurement Multiplicity ..........         3.3 Low
PLAT951_ALERT_5_G Calculated (ThMax) and CIF-Reported Kmax Differ           2 Units
PLAT965_ALERT_2_G The SHELXL WEIGHT Optimisation has not Converged     Please Check
PLAT967_ALERT_5_G Note: Two-Theta Cutoff Value in Embedded .res ..       50.0 Degree
```

---

0 **ALERT level A** = Most likely a serious problem - resolve or explain
0 **ALERT level B** = A potentially serious problem, consider carefully
15 **ALERT level C** = Check. Ensure it is not caused by an omission or oversight
41 **ALERT level G** = General information/check it is not something unexpected

2 ALERT type 1 CIF construction/syntax error, inconsistent or missing data
16 ALERT type 2 Indicator that the structure model may be wrong or deficient
12 ALERT type 3 Indicator that the structure quality may be low
23 ALERT type 4 Improvement, methodology, query or suggestion
3 ALERT type 5 Informative message, check

It is advisable to attempt to resolve as many as possible of the alerts in all categories. Often the minor alerts point to easily fixed oversights, errors and omissions in your CIF or refinement strategy, so attention to these fine details can be worthwhile. In order to resolve some of the more serious problems it may be necessary to carry out additional measurements or structure refinements. However, the purpose of your study may justify the reported deviations and the more serious of these should normally be commented upon in the discussion or experimental section of a paper or in the "special_details" fields of the CIF. checkCIF was carefully designed to identify outliers and unusual parameters, but every test has its limitations and alerts that are not important in a particular case may appear. Conversely, the absence of alerts does not guarantee there are no aspects of the results needing attention. It is up to the individual to critically assess their own results and, if necessary, seek expert advice.

Publication of your CIF in IUCr journals

A basic structural check has been run on your CIF. These basic checks will be run on all CIFs submitted for publication in IUCr journals (*Acta Crystallographica*, *Journal of Applied Crystallography*, *Journal of Synchrotron Radiation*); however, if you intend to submit to *Acta Crystallographica Section C* or *E* or *IUCrData*, you should make sure that full publication checks are run on the final version of your CIF prior to submission.

Publication of your CIF in other journals

Please refer to the *Notes for Authors* of the relevant journal for any special instructions relating to CIF submission.

PLATON version of 06/07/2023; check.def file version of 30/06/2023

Datablock 20191011-CZL-PY-2-2 - ellipsoid plot

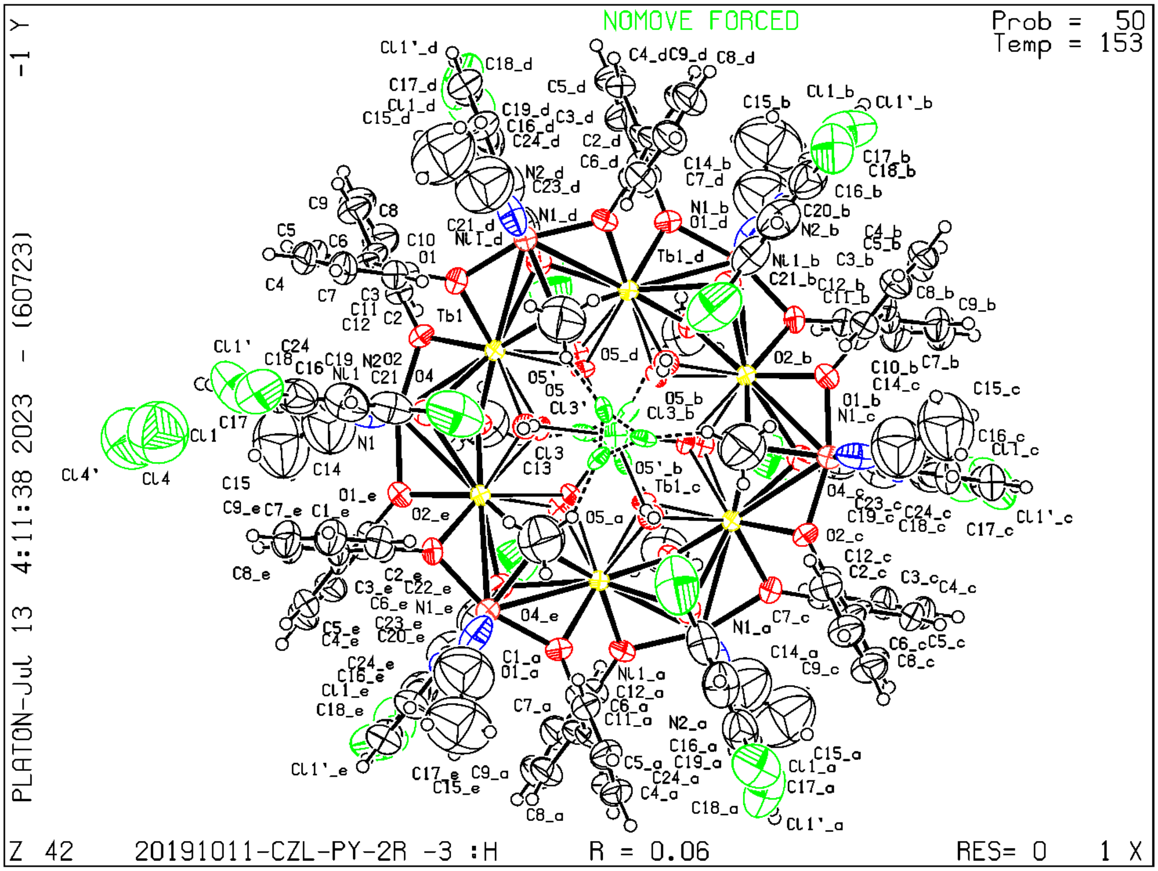

Supplement: Supplementary file 1 [file molecules-28-05906-s001.zip › checkcif of 3.pdf]
