# Supplementary material for: Russian Doll-like 3d–4f Cluster Wheels with Slow Relaxation of Magnetization
Source: Molecules. 2023 Aug 6;28(15):5906. doi: 10.3390/molecules28155906 (PMC10421525; doi:10.3390/molecules28155906)
Supplement: Supplementary file 1 [file molecules-28-05906-s001.zip › Supplementary materials of molecules-2547928.pdf]

Supplementary Materials

Russian Doll-like 3d–4f Cluster Wheels with Slow Relaxation of Magnetization †

Lan Liu [1,‡], Panpan Yang [1,‡], Zhihui Qiu [1,*], Kai Wang [2,*], Dongcheng Liu [1], Yuning Liang [1], Huancheng Hu [1], Huahong Zou [1], Fupei Liang [1,2] and Zilu Chen [1,*]

1 School of Chemistry and Pharmaceutical Sciences, Guangxi Normal University, Guilin 541004, China
2 Guangxi Key Laboratory of Electrochemical and Magnetochemical Functional Materials, College of Chemistry and Bioengineering, Guilin University of Technology, Guilin 541004, China
* Correspondence: zhihuiqiu@mailbox.gxnu.edu.cn (Z.Q.); kaiwang@glut.edu.cn (K.W.); zlchen@mailbox.gxnu.edu.cn (Z.C.)
† Electronic supplementary information (ESI) available: General materials and methods, crystallography details, PXRD patterns, additional figures for structural and magnetic properties. CCDC 2252491-2252493 respectively correspond to **1**–**3**.

‡ These authors contributed equally to this work.

Contents

1. Experimental Section S3

2. Supporting Figures S4

3. Supporting Tables S11

Experimental Section

X-ray Data Collection and Structure Refinement

The diffraction intensity data of these complexes were collected on SuperNova (**1** and **3**) and Synergy Custom DW (**2**) diffractometers. Their single-crystal structures were solved by the direct method using SHELXS and then refined using the program of SHELXL. All non-hydrogen atoms (C, N, O, Cl, Co, Ni, Dy, Tb) in the complexes were located from difference Fourier maps and subsequently refined with anisotropic displacement parameters. The central metal ion (Dy(III) for **1** and **2**, and Tb(III) for **3**) is disordered over two symmetry-related positions in an occupancy ratio of 0.5 : 0.5. The coordinated Cl^- and μ_3-OH^- ligands in the three titled complexes are both disordered. In addition, the CH_3CN ligands, the two Cl atoms on $(L^2)^-$ ligand, and the free Cl^- ion in **2** are also modelled as disorder. In **3**, one Cl atom on $(L^2)^-$ ligand, and the free Cl^- ion are also modelled as disorder. The H atoms attached on C atoms were refined at the geometrical sites. The H atoms attached on the O atoms were located on a difference Fourier map. The detailed parameters for these structures are listed in Table S1. The selected bond lengths and bond angles of the complexes are presented in Table S2-S4, respectively, in the Supporting information.

Supporting Figures

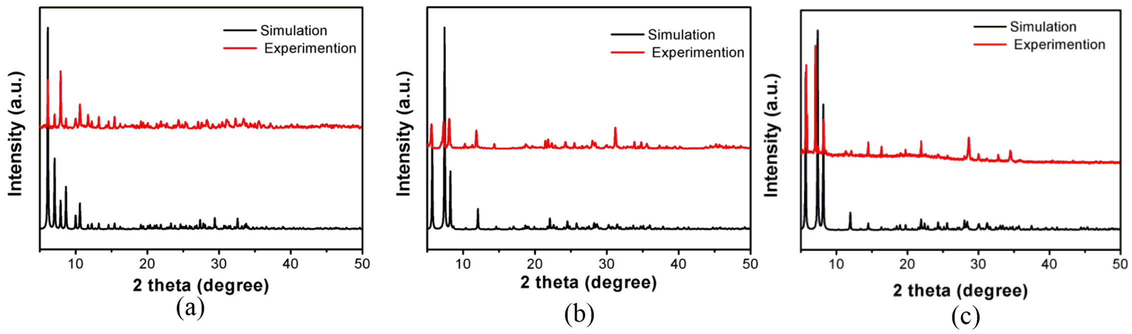

Figure S1. (a), (b), (c) correspond to the powder diffraction patterns of complexes **1-3**, respectively.

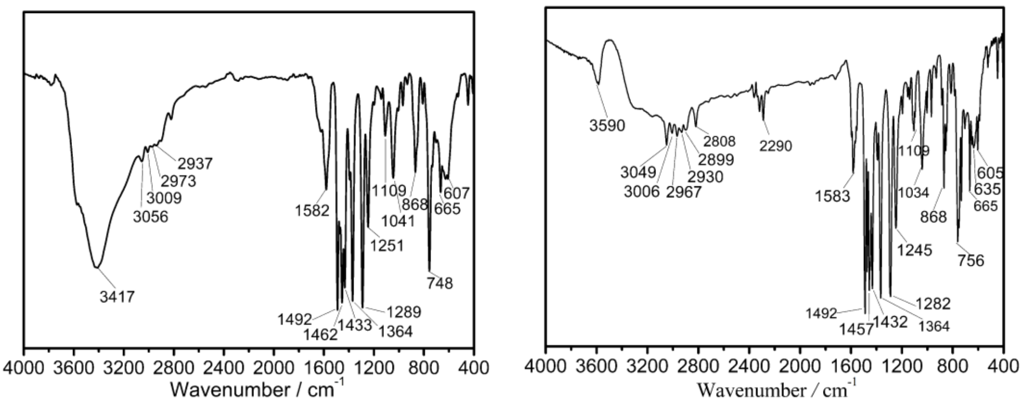

Figure S2. Infrared spectra of complexes **2** (left) and **3** (right).

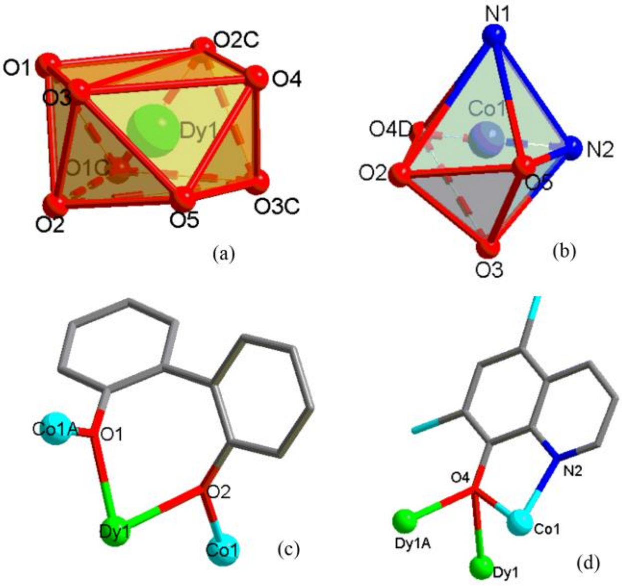

Figure S3. (a) Coordination mode diagram of Dy(III); (b) Coordination mode diagram of Co(II); (c) Ligand $(L^1)^{2-}$ coordination pattern diagram; (d) Coordination pattern of ligand $(L^2)^{-}$.

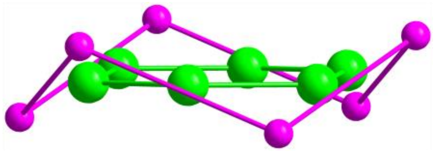

Figure S4. Metal skeleton of Co_6Dy_6 metallacrown ether in **1**.

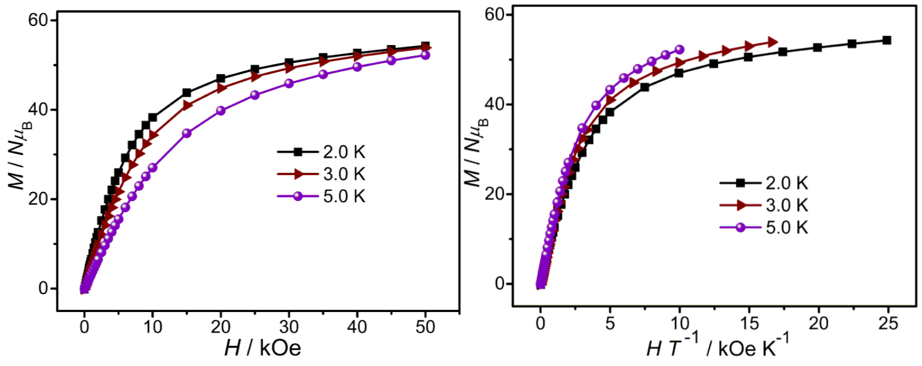

(a) (b)

Figure S5. *M-H* (a) and *M-HT*$^{-1}$ (b) curves of **1**.

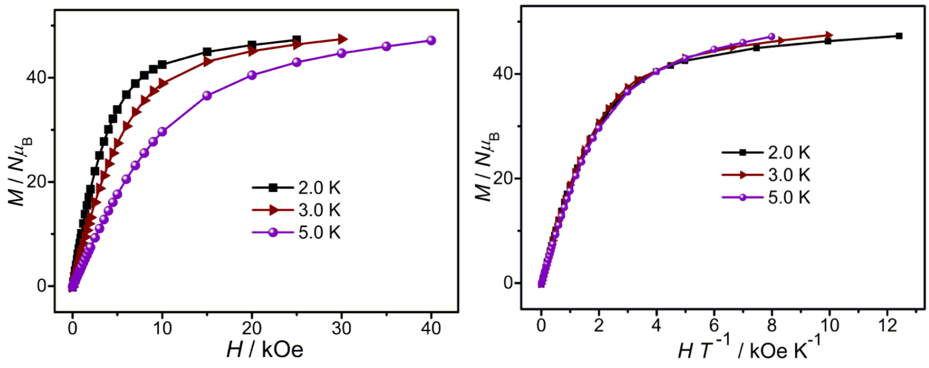

(a) (b)

Figure S6. *M-H* (a) and *M-HT*$^{-1}$ (b) curves of **2**.

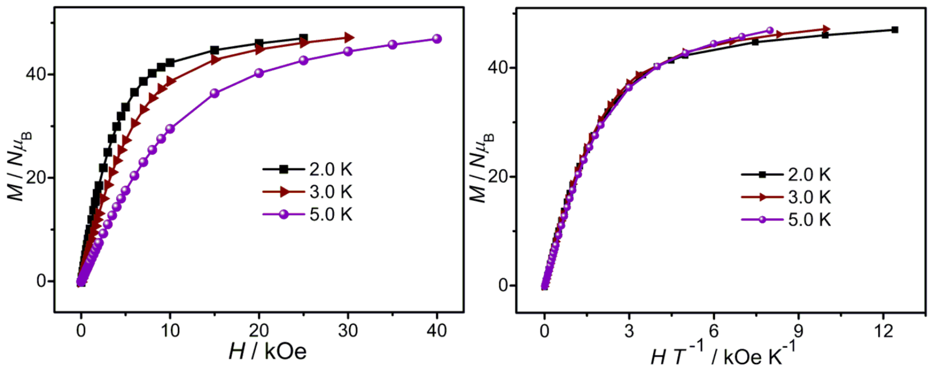

(a) (b)

Figure S7. *M-H* (a) and *M-HT*$^{-1}$ (b) curves of **3**.

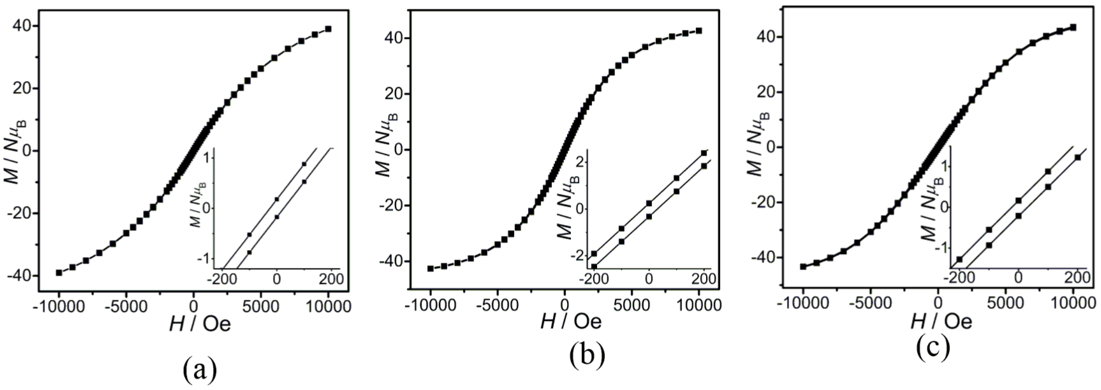

Figure S8. Hysteresis curves measured at 2 K for **1** (a), **2** (b), **3** (c) with the scan rate of 200 Oe/s.

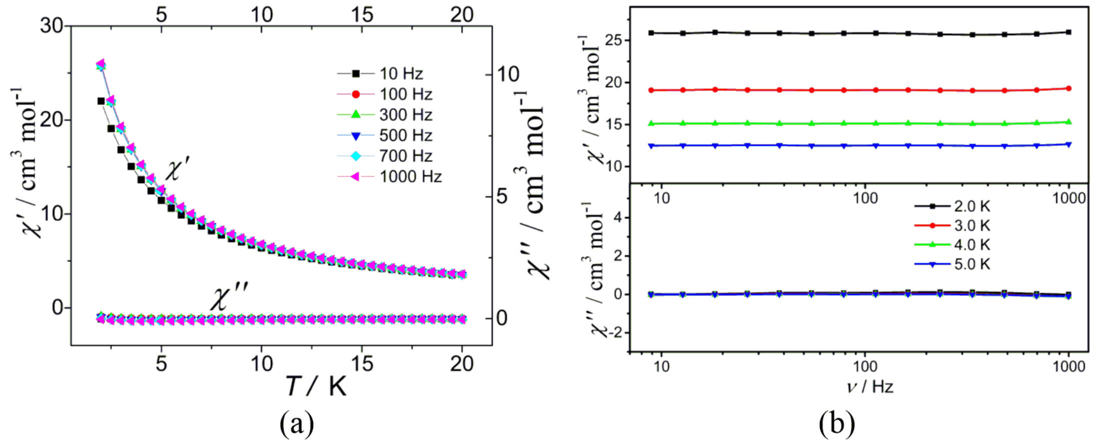

Figure S9. Temperature-dependent (a) and frequency-dependent (b) ac magnetic susceptibilities under zero dc field for **1**.

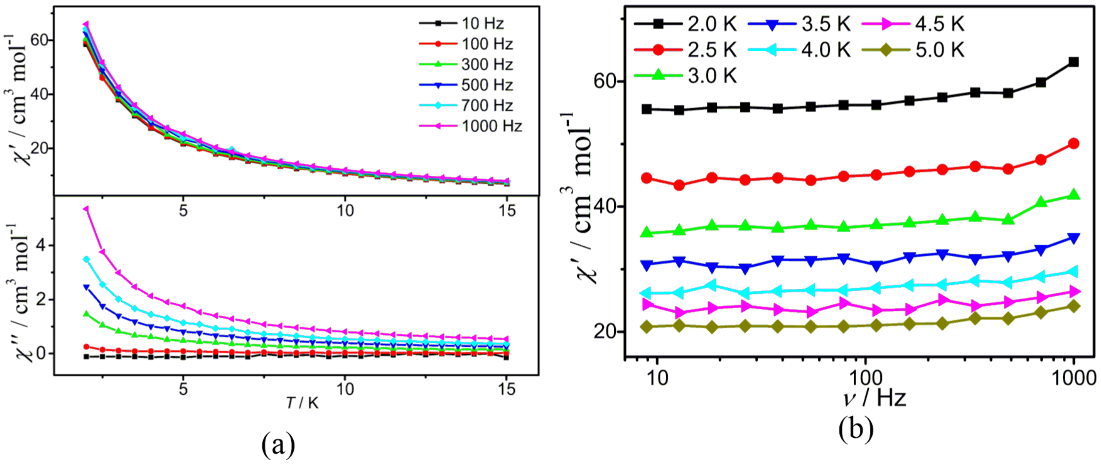

Figure S10. Temperature-dependent (a) and frequency-dependent (b) ac magnetic susceptibilities under zero dc field for **2**.

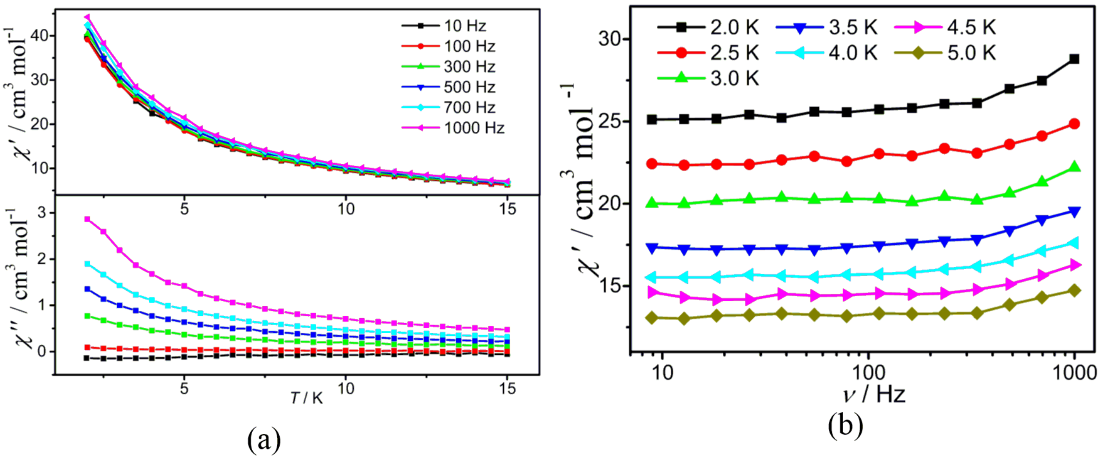

Figure S11. Temperature-dependent (a) and frequency-dependent (b) ac magnetic susceptibilities under zero dc field for **3**.

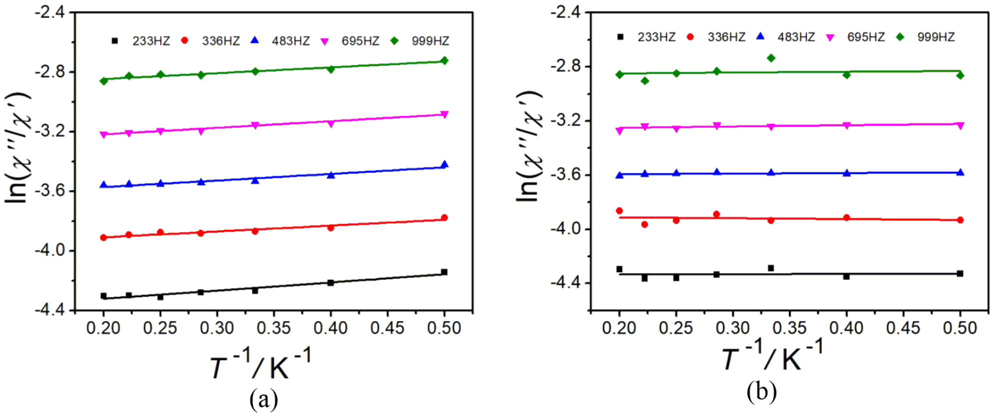

Figure S12. (a) and (b) correspond to ln(χ''/χ') versus 1/T plots for complexes **2** and **3**, respectively.

Supporting Tables

Table S1. Crystal data and structural refining parameters for **1-3.**

complex	**1**	**2**	**3**
Formula	$C_{144}H_{120}N_{12}O_{33}Cl_{15}Co_6Dy_7$	$C_{144}H_{114}N_{12}O_{30}Cl_{15}Ni_6Dy_7$	$C_{144}H_{114}N_{12}O_{30}Cl_{15}Ni_6Tb_7$
Fw	4569.34	4513.98	4488.92
T / K	153(2)	153(2)	153(2)
λ / Å	0.71073	0.71073	0.71073
Crystal system	Cubic	Trigonal	Trigonal
Space group	*P*a-3	*R*-3	*R*-3
a / Å	25.0221(2)	20.4123(4)	20.5601(7)
b / Å	25.0221(2)	20.4123(4)	20.5601(7)
c / Å	25.0221(2)	32.2304(8)	32.526(3)
α / °	90.00	90.00	90.00
β / °	90.00	90.00	90.00
γ / °	90.00	120.00	120.00
V / Å^3	15666.5(4)	11630.1(5)	11907.2(13)
Z	4	3	3
D_c / g cm^{-3}	1.937	1.934	1.878
μ / mm^{-1}	4.240	4.368	4.090
F(000)	8844	6561	6540
θ / °	1.820 to 25.007	1.995 to 25.011	3.393 to 25.019
Reflns collected	23685	15007	15569
Reflns unique	4622	4561	4671
R_{int}	0.0331	0.0275	0.0435
GOOF on F^2	1.069	1.105	1.038
R_1 [$I > 2\sigma(I)$]	0.0377	0.0489	0.0595
wR_2 [$I > 2\sigma(I)$)]	0.1020	0.1313	0.1435
R_1 (all data)	0.0475	0.0549	0.0796
wR_2 (all data)	0.1108	0.1383	0.1639

Table S2 Selected bond lengths (Å) and angles (º) for complex **1**

Co1-O2	2.053(4)	O1-Dy1-O2	75.83(15)	Dy2C-Dy2-O5'E	100.7(3)
Co1-O1A	2.061(4)	O1-Dy1-O5	132.6(3)	O5'D-Dy2-O5'E	116.66(18)
Co1-N1	2.065(6)	O2-Dy1-O5	133.1(3)	Dy2C-Dy2-O5'	100.7(3)
Co1-N2	2.099(6)	O1-Dy1-O3	145.23(15)	O5'D-Dy2-O5'	116.66(18)
Co1-O3	2.110(4)	O2-Dy1-O3	71.68(15)	O5'E-Dy2-O5'	116.66(18)
Co1-O4	2.154(4)	O5-Dy1-O3	67.9(3)	Dy2C-Dy2-O5B	51.9(3)
Dy1-O1	2.265(4)	O1-Dy1-O3B	69.72(15)	O5D-Dy2-O5B	136.3(2)
Dy1-O2	2.277(4)	O2-Dy1-O3B	142.68(14)	O5E-Dy2-O5B	63.1(3)
Dy1-O5	2.338(11)	O5-Dy1-O3B	82.5(3)	O5-Dy2-O5B	63.1(3)
Dy1-O3	2.345(4)	O3-Dy1-O3B	144.7(2)	Dy2C-Dy2-O5C	51.9(3)
Dy1-O3B	2.364(4)	O1-Dy1-O5B	133.3(3)	O5D-Dy2-O5C	63.1(3)
Dy1-O5B	2.372(11)	O2-Dy1-O5B	136.8(3)	O5E-Dy2-O5C	63.1(3)
Dy1-O5'	2.377(11)	O5-Dy1-O5B	56.6(5)	O5-Dy2-O5C	136.3(2)
Dy1-O5'B	2.442(11)	O3-Dy1-O5B	80.5(3)	O5B-Dy2-O5C	85.9(4)
Dy1-O4B	2.513(4)	O3B-Dy1-O5B	67.0(3)	Dy2C-Dy2-O5A	51.9(3)
Dy1-O4	2.573(4)	O1-Dy1-O5'	116.9(3)	O5D-Dy2-O5A	63.1(3)
Dy2-O5D	1.852(10)	O2-Dy1-O5'	130.1(3)	O5E-Dy2-O5A	136.3(2)
Dy2-O5E	1.852(10)	O3-Dy1-O5'	77.1(3)	O5-Dy2-O5A	63.1(3)
Dy2-O5	1.852(10)	O3B-Dy1-O5'	79.8(3)	O5B-Dy2-O5A	85.9(4)
Dy2-O5'D	2.253(10)	O1-Dy1-O5'B	132.4(3)	O5C-Dy2-O5A	85.9(4)
Dy2-O5'E	2.253(10)	O2-Dy1-O5'B	121.4(3)	O5D-Dy2-Cl3	95.6(4)
Dy2-O5'	2.253(10)	O3-Dy1-O5'B	76.9(3)	O5E-Dy2-Cl3	95.6(4)
Dy2-O5B	2.341(11)	O3B-Dy1-O5'B	75.5(3)	O5-Dy2-Cl3	95.6(4)
Dy2-O5C	2.341(11)	O5'-Dy1-O5'B	86.7(5)	O5B-Dy2-Cl3	128.1(3)
Dy2-O5A	2.341(11)	O1-Dy1-O4B	74.38(15)	O5C-Dy2-Cl3	128.1(3)
Dy2-Cl3	2.598(15)	O2-Dy1-O4B	92.16(15)	O5A-Dy2-Cl3	128.1(3)
Dy2-Cl3'	2.701(8)	O5-Dy1-O4B	127.6(3)	Dy2C-Dy2-Cl3'	165.9(3)
O1-Co1B	2.061(4)	O3-Dy1-O4B	118.39(14)	O5'D-Dy2-Cl3'	72.4(4)
O3-Dy1A	2.364(4)	O3B-Dy1-O4B	65.39(14)	O5'E-Dy2-Cl3'	72.7(4)
O4-Dy1A	2.513(4)	O5B-Dy1-O4B	72.6(3)	O5'-Dy2-Cl3'	93.5(4)
O5-Dy2C	2.342(11)	O5'-Dy1-O4B	137.2(3)	Co1B-O1-Dy1	94.22(16)
O5-Dy1A	2.372(11)	O5'B-Dy1-O4B	61.9(3)	Co1-O2-Dy1	95.51(16)
O5'-Dy1A	2.442(11)	O1-Dy1-O4	92.90(15)	Co1-O3-Dy1	92.03(16)
O2-Co1-O1A	159.08(17)	O2-Dy1-O4	69.95(14)	Co1-O3-Dy1A	90.13(15)
O2-Co1-N1	95.8(2)	O5-Dy1-O4	72.0(3)	Dy1-O3-Dy1A	102.41(17)
O1A-Co1-N1	96.2(2)	O3-Dy1-O4	64.67(14)	Co1-O4-Dy1A	85.31(15)
O2-Co1-N2	99.5(2)	O3B-Dy1-O4	124.72(14)	Co1-O4-Dy1	85.04(15)
O1A-Co1-N2	96.5(2)	O5B-Dy1-O4	126.0(3)	Dy1A-O4-Dy1	92.38(14)
N1-Co1-N2	94.8(2)	O5'-Dy1-O4	61.8(3)	Dy2-O5-Dy1	126.4(5)
O2-Co1-O3	81.09(16)	O5'B-Dy1-O4	134.1(3)	Dy2-O5-Dy2C	43.7(2)
O1A-Co1-O3	78.78(16)	O4B-Dy1-O4	160.39(15)	Dy1-O5-Dy2C	107.4(4)
N1-Co1-O3	109.4(2)	Dy2C-Dy2-O5D	84.4(4)	Dy2-O5-Dy1A	125.9(5)

N2-Co1-O3	155.6(2)	Dy2C-Dy2-O5E	84.4(4)	Dy1-O5-Dy1A	102.4(4)
O2-Co1-O4	83.08(17)	O5D-Dy2-O5E	119.05(12)	Dy2C-O5-Dy1A	105.2(4)
O1A-Co1-O4	86.72(17)	Dy2C-Dy2-O5	84.4(4)	Dy2-O5'-Dy1	108.0(4)
N1-Co1-O4	173.9(2)	O5D-Dy2-O5	119.05(12)	Dy2-O5'-Dy1A	106.8(4)
N2-Co1-O4	79.47(19)	O5E-Dy2-O5	119.06(12)	Dy1-O5'-Dy1A	99.2(4)
O3-Co1-O4	76.40(17)	Dy2C-Dy2-O5'D	100.7(3)		

Symmetry transformations used to generate equivalent atoms: A) *y* + 1/2, *z*, -*x* + 1/2; B) -*z* + 1/2, *x* - 1/2, *y*; C) -*x* + 1, -*y*, -*z*; D) *z* + 1/2, -*x* + 1/2, -*y*; E) -*y* + 1/2, -*z*, *x* - 1/2

Table S3 Selected bond lengths (Å) and angles (°) for complex **2**

Dy1-O2	2.250(5)	O3A-Dy1-O5B	81.4(3)	O5C-Dy2-O5E	56.8(2)
Dy1-O1	2.251(5)	O2-Dy1-O3	69.71(19)	O5D-Dy2-O5E	56.8(2)
Dy1-O5	2.322(8)	O1-Dy1-O3	143.63(18)	O5-Dy2-O5E	124.8(3)
Dy1-O3A	2.340(6)	O5-Dy1-O3	83.4(3)	O5A-Dy2-O5E	84.3(3)
Dy1-O5B	2.343(9)	O3A-Dy1-O3	143.1(3)	O5B-Dy2-O5E	84.3(3)
Dy1-O3	2.386(6)	O5B-Dy1-O3	63.8(3)	O5C-Dy2-Cl3C	98.1(4)
Dy1-O4	2.451(5)	O2-Dy1-O4	73.00(17)	O5D-Dy2-Cl3C	98.1(4)
Dy1-O5'	2.475(10)	O1-Dy1-O4	92.19(18)	O5-Dy2-Cl3C	121.3(4)
Dy1-O5'B	2.533(10)	O5-Dy1-O4	127.4(3)	O5A-Dy2-Cl3C	135.3(3)
Dy1-O4A	2.592(5)	O3A-Dy1-O4	120.43(19)	O5B-Dy2-Cl3C	135.4(3)
Dy1-Ni1	3.1578(10)	O5B-Dy1-O4	75.8(3)	O5E-Dy2-Cl3C	114.0(3)
Dy1-Ni1A	3.2016(10)	O3-Dy1-O4	64.44(19)	N2-Ni1-O2	96.87(19)
Dy1-H(51)	2.4715	O2-Dy1-O5'	110.9(3)	N2-Ni1-O3	157.1(3)
Dy2-O5C	1.896(9)	O1-Dy1-O5'	130.3(3)	O2-Ni1-O3	80.2(2)
Dy2-O5D	1.896(9)	O3A-Dy1-O5'	80.5(3)	N2-Ni1-O1B	95.9(2)
Dy2-O5	1.896(9)	O3-Dy1-O5'	77.1(3)	O2-Ni1-O1B	159.7(2)
Dy2-Dy2E	2.010(3)	O4-Dy1-O5'	137.5(3)	O3-Ni1-O1B	81.9(2)
Dy2-Cl3'	2.09(3)	O2-Dy1-O5'B	133.9(2)	N2-Ni1-N1'	95.3(7)
Dy2-O5'D	2.282(10)	O1-Dy1-O5'B	117.1(3)	O2-Ni1-N1'	96.7(6)
Dy2-O5'C	2.282(10)	O3A-Dy1-O5'B	73.7(3)	O3-Ni1-N1'	107.6(7)
Dy2-O5'	2.282(9)	O3-Dy1-O5'B	78.4(3)	O1B-Ni1-N1'	97.7(7)
Dy2-O5A	2.351(10)	O4-Dy1-O5'B	63.3(2)	N2-Ni1-O4	80.6(2)
Dy2-O5B	2.351(10)	O5'-Dy1-O5'B	92.7(5)	O2-Ni1-O4	84.6(2)
Dy2-O5E	2.351(10)	O2-Dy1-O4A	93.99(17)	O3-Ni1-O4	76.5(3)
Dy2-Cl3C	2.741(10)	O1-Dy1-O4A	68.53(16)	O1B-Ni1-O4	82.1(2)
Ni1-N2	2.037(4)	O5-Dy1-O4A	73.4(3)	N1'-Ni1-O4	175.9(7)
Ni1-O2	2.057(5)	O3A-Dy1-O4A	62.82(18)	N2-Ni1-N1	102.7(8)
Ni1-O3	2.061(5)	O5B-Dy1-O4A	124.2(2)	O2-Ni1-N1	94.7(7)
Ni1-O1B	2.069(5)	O3-Dy1-O4A	127.19(19)	O3-Ni1-N1	100.2(8)
Ni1-N1'	2.08(2)	O4-Dy1-O4A	159.05(18)	O1B-Ni1-N1	97.8(8)
Ni1-O4	2.106(5)	O5'-Dy1-O4A	62.1(2)	O4-Ni1-N1	176.7(8)
Ni1-N1	2.29(3)	O5'B-Dy1-O4A	132.1(2)	Ni1A-O1-Dy1	95.56(19)
Ni1-Dy1B	3.2017(10)	O5C-Dy2-O5D	112.7(3)	Ni1-O2-Dy1	94.20(19)

O1-Ni1A	2.069(5)	O5C-Dy2-O5	112.7(3)	Ni1-O3-Dy1	90.2(2)
O3-Dy1B	2.340(6)	O5D-Dy2-O5	112.7(3)	Dy1B-O3-Dy1	101.9(3)
O4-Dy1B	2.592(5)	Cl3'-Dy2-O5'D	79.4(3)	Ni1-O4-Dy1	87.39(19)
O5-Dy1A	2.342(9)	Cl3'-Dy2-O5'C	79.4(3)	Ni1-O4-Dy1B	85.26(19)
O5-Dy2E	2.351(10)	O5'D-Dy2-O5'C	116.71(18)	Dy1-O4-Dy1B	93.41(19)
O5'-Dy1A	2.533(10)	Cl3'-Dy2-O5'	79.4(3)	Dy2-O5-Dy1	127.7(4)
O2-Dy1-O1	77.15(18)	O5'D-Dy2-O5'	116.71(18)	Dy2-O5-Dy1A	128.3(4)
O2-Dy1-O5	134.4(3)	O5'C-Dy2-O5'	116.71(18)	Dy1-O5-Dy1A	103.8(3)
O1-Dy1-O5	132.0(3)	O5C-Dy2-O5A	124.8(3)	Dy2-O5-Dy2E	55.2(3)
O2-Dy1-O3A	146.69(18)	O5D-Dy2-O5A	56.8(2)	Dy1-O5-Dy2E	109.7(4)
O1-Dy1-O3A	72.26(19)	O5-Dy2-O5A	56.8(2)	Dy1A-O5-Dy2E	107.8(4)
O5-Dy1-O3A	64.8(3)	O5C-Dy2-O5B	56.8(2)	Dy2-O5'-Dy1	105.6(4)
O2-Dy1-O5B	131.7(2)	O5D-Dy2-O5B	124.8(3)	Dy2-O5'-Dy1A	104.9(4)
O1-Dy1-O5B	140.1(3)	O5-Dy2-O5B	56.8(2)	Dy1-O5'-Dy1A	94.3(4)
O5-Dy1-O5B	52.4(4)	O5A-Dy2-O5B	84.3(3)		

Symmetry transformations used to generate equivalent atoms: A) *y* + 1⁄3, -*x* + *y* + 2⁄3, -*z* + 2⁄3; B) *x* - *y* + 1⁄3, *x* - 1⁄3, -*z* + 2⁄3; C) -*y* + 1, *x* - *y*, *z*; D) -*x* + *y* + 1, -*x* + 1, *z*; E) -*x* + 4⁄3, -*y* + 2⁄3, -*z* + 2⁄3.

Table S4 Selected bond lengths (Å) and angles (º) for complex **3**

Tb1-O2	2.254(6)	O3A-Tb1-O5B	81.9(3)	O5-Tb2-O5B	57.5(3)
Tb1-O1	2.262(6)	O2-Tb1-O3	69.9(2)	O5A-Tb2-O5B	84.5(4)
Tb1-O5	2.330(11)	O1-Tb1-O3	143.6(2)	O5C-Tb2-O5E	57.5(3)
Tb1-O3A	2.351(7)	O5-Tb1-O3	83.2(3)	O5D-Tb2-O5E	57.5(3)
Tb1-O5B	2.364(11)	O3A-Tb1-O3	143.6(3)	O5-Tb2-O5E	126.0(3)
Tb1-O3	2.401(7)	O5B-Tb1-O3	64.0(3)	O5A-Tb2-O5E	84.5(4)
Tb1-O5'	2.469(13)	O2-Tb1-O5'	112.9(4)	O5B-Tb2-O5E	84.5(4)
Tb1-O4	2.482(7)	O1-Tb1-O5'	130.0(3)	O5C-Tb2-Cl3C	96.8(5)
Tb1-O5'B	2.519(13)	O3A-Tb1-O5'	79.6(4)	O5D-Tb2-Cl3C	97.1(5)
Tb1-O4A	2.596(7)	O3-Tb1-O5'	77.6(3)	O5-Tb2-Cl3C	120.2(5)
Tb1-Ni1	3.1758(13)	O2-Tb1-O4	71.9(2)	O5A-Tb2-Cl3C	135.4(4)
Tb1-Ni1A	3.2112(13)	O1-Tb1-O4	92.8(2)	O5B-Tb2-Cl3C	135.1(4)
Tb2-O5C	1.908(11)	O5-Tb1-O4	127.3(3)	O5E-Tb2-Cl3C	113.8(4)
Tb2-O5D	1.908(11)	O3A-Tb1-O4	121.3(2)	N2-Ni1-O2	96.6(3)
Tb2-O5	1.908(11)	O5B-Tb1-O4	75.1(3)	N2-Ni1-O1B	96.0(3)
Tb2-Tb2E	1.987(4)	O3-Tb1-O4	63.9(2)	O2-Ni1-O1B	159.9(3)
Tb2-Cl3'	2.11(4)	O5'-Tb1-O4	137.1(3)	N2-Ni1-O3	157.5(3)
Tb2-O5'D	2.298(13)	O2-Tb1-O5'B	132.7(3)	O2-Ni1-O3	80.6(2)
Tb2-O5'C	2.298(13)	O1-Tb1-O5'B	118.1(4)	O1B-Ni1-O3	81.7(3)
Tb2-O5'	2.298(13)	O3A-Tb1-O5'B	75.0(3)	N2-Ni1-O4	80.8(3)
Tb2-O5A	2.374(13)	O3-Tb1-O5'B	77.7(4)	O2-Ni1-O4	84.3(3)
Tb2-O5B	2.374(13)	O5'-Tb1-O5'B	91.8(6)	O1B-Ni1-O4	82.4(3)
Tb2-O5E	2.374(13)	O4-Tb1-O5'B	63.1(3)	O3-Ni1-O4	76.7(3)

Tb2-Cl3C	2.716(14)	O2-Tb1-O4A	94.2(2)	N2-Ni1-N1	99.1(4)
Ni1-N2	2.031(6)	O1-Tb1-O4A	68.4(2)	O2-Ni1-N1	95.7(3)
Ni1-O2	2.059(6)	O5-Tb1-O4A	73.5(3)	O1B-Ni1-N1	97.6(3)
Ni1-O1B	2.070(6)	O3A-Tb1-O4A	62.8(2)	O3-Ni1-N1	103.5(5)
Ni1-O3	2.071(7)	O5B-Tb1-O4A	125.0(3)	O4-Ni1-N1	179.8(4)
Ni1-O4	2.096(7)	O3-Tb1-O4A	126.8(2)	Ni1A-O1-Tb1	95.6(2)
Ni1-N1	2.144(13)	O5’-Tb1-O4A	62.2(3)	Ni1-O2-Tb1	94.7(2)
Ni1-Tb1B	3.2112(13)	O4-Tb1-O4A	159.2(2)	Ni1-O3-Tb1B	92.9(3)
O1-Ni1A	2.070(6)	O5’B-Tb1-O4A	133.0(3)	Ni1-O3-Tb1	90.2(2)
O3-Tb1B	2.351(7)	O5C-Tb2-O5D	113.6(3)	Tb1B-O3-Tb1	102.1(3)
O4-Tb1B	2.596(7)	O5C-Tb2-O5	113.6(3)	Ni1-O4-Tb1	87.4(3)
O5-Tb1A	2.364(11)	O5D-Tb2-O5	113.6(3)	Ni1-O4-Tb1B	85.7(2)
O5-Tb2E	2.374(13)	Cl3’-Tb2-O5’D	80.1(4)	Tb1-O4-Tb1B	93.4(2)
O5’-Tb1A	2.518(13)	Cl3’-Tb2-O5’C	80.1(4)	Tb2-O5-Tb1	128.1(5)
O2-Tb1-O1	76.5(2)	O5’D-Tb2-O5’C	117.1(2)	Tb2-O5-Tb1A	127.6(6)
O2-Tb1-O5	135.0(4)	Cl3’-Tb2-O5’	80.1(4)	Tb1-O5-Tb1A	103.9(4)
O1-Tb1-O5	132.1(3)	O5’D-Tb2-O5’	117.1(2)	Tb2-O5-Tb2E	54.0(3)
O2-Tb1-O3A	146.0(2)	O5’C-Tb2-O5’	117.1(2)	Tb1-O5-Tb2E	109.4(5)
O1-Tb1-O3A	71.9(2)	O5C-Tb2-O5A	126.0(3)	Tb1A-O5-Tb2E	107.3(5)
O5-Tb1-O3A	65.3(4)	O5D-Tb2-O5A	57.5(3)	Tb2-O5’-Tb1	106.3(5)
O2-Tb1-O5B	131.6(3)	O5-Tb2-O5A	57.5(3)	Tb2-O5’-Tb1A	105.6(5)
O1-Tb1-O5B	139.8(4)	O5C-Tb2-O5B	57.5(3)	Tb1-O5’-Tb1A	95.7(5)
O5-Tb1-O5B	53.1(5)	O5D-Tb2-O5B	126.0(3)		

Symmetry transformations used to generate equivalent atoms: A) *y* + 1/3, -*x* + *y* + 2/3, -*z* + 2/3; B) *x* – *y* + 1/3, *x* - 1/3, -*z* + 2/3; C) –*y* + 1, *x* - *y*, *z*; D) –*x* + *y* + 1, -*x* + 1, *z*; E) –*x* + 4/3, -*y* + 2/3, -*z* + 2/3.
